# Immunoregulatory Roles of Tumor‐Originated Pericytes Identified by Single‐Cell Analysis in Glioblastoma

**DOI:** 10.1002/advs.202511856

**Published:** 2025-09-26

**Authors:** Cuiying Chu, Fangzhen Li, Zhiwen Zhang, Qiuhong Zhu, Hong Yan, Mengdan Cheng, Huipeng Wang, Lei Cheng, Zhe Zhang, Xingjiang Yu, Jianghong Man, Wei Wang, Dongxue Li, Xiu‐wu Bian, Hai‐bo Wu, Aili Zhang, Wenchao Zhou

**Affiliations:** ^1^ Department of Pathology, Centre for Leading Medicine and Advanced Technologies of IHM The First Affiliated Hospital of USTC Division of Life Sciences and Medicine University of Science and Technology of China Hefei 230036 China; ^2^ Intelligent Pathology Institute The First Affiliated Hospital of USTC Division of Life Sciences and Medicine University of Science and Technology of China Hefei 230036 China; ^3^ Institute of Children's Health Henan Academy of Medical Sciences Zhengzhou 451162 China; ^4^ Department of Neurosurgery The Affiliated Hospital of Qingdao University Qingdao 266000 China; ^5^ Basic Medical College Qingdao University Qingdao 266073 China; ^6^ Department of Histology and Embryology School of Basic Medicine Tongji Medical College Hubei Key Laboratory of Drug Target Research and Pharmacodynamic Evaluation Huazhong University of Science and Technology Wuhan 430030 China; ^7^ Nanhu Laboratory National Center of Biomedical Analysis Beijing 100850 China; ^8^ Department of Neurosurgery the First Affiliated Hospital of USTC Division of Life Sciences and Medicine University of Science and Technology of China Hefei 230036 China; ^9^ Institute of Pathology and Southwest Cancer Center Southwest Hospital Third Military Medical University (Army Medical University) Chongqing 400038 China; ^10^ Research Center for Translational Medicine The Second Affiliated Hospital of Anhui Medical University Hefei 230601 China; ^11^ Department of Cell Biology School of Life Science Anhui Medical University Hefei Anhui 230012 China

**Keywords:** glioblastoma, macrophage, pericyte, tumor immunology, vasculature

## Abstract

Pericytes as critical vascular support cells not only keep the integrity of blood–brain barrier but also play profound roles in brain tumors. Yet the origin and functional heterogeneity of pericytes in the most common malignant brain tumor glioblastoma (GBM) remain unclear. Here, single‐cell RNA‐sequencing (scRNA‐seq) is performed on CD146^+^ pericytes from human primary GBMs. Tumor‐ and normal‐originated pericytes (T‐PCs and N‐PCs) that have distinctive cell‐intrinsic features and intercellular communications with endothelial and immune cells are identified. Bioinformatic analyses on integrated in‐house and public scRNA‐seq data have found a T‐PC metacluster marked by CD44 closely associated with tumor‐associated macrophages (TAMs). The CD44^High^ pericytes are detected in human GBM samples and glioma‐stem‐cell (GSC)‐derived pericytes. Coimplantation of GSC‐derived CD44^High^ pericytes promotes M2 polarization of TAMs and growth of orthotopic GBMs. In summary, this study unravels the existence of T‐PCs and N‐PCs in GBMs, analyzes their functional heterogeneity, and unravels the immunoregulatory roles of CD44^High^ pericytes. These discoveries help to gain insights into brain tumor vasculature and inspire therapeutic strategies targeting vessels and TAMs for GBM treatment.

## Graphical abstract

The origin and functional heterogeneity of pericytes in glioblastoma (GBM) remain unclear. This study identifies tumor‐originated pericytes (T‐PCs) and normal‐originated pericytes as two distinctive populations in human GBM using single‐cell RNA‐sequencing. A T‐PC metacluster marked by CD44 is shown to promote M2 polarization of tumor‐associated macrophages and tumor growth. These insights into brain tumor vasculature may inspire therapeutic strategies.

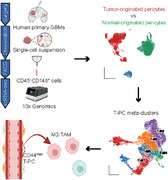

## 1 Introduction

Pericytes are mural cells located along microvasculature in almost all organs. As vascular support cell population, pericytes participate in vessel formation, maintenance of vessel integrity, and control of blood flow.^[^ ^1^, ^2^ ^]^ Pericytes are especially important for the brain vasculature, in which they function as indispensable components of the neurovascular units required for the blood–brain barrier (BBB) and homeostasis of the central nervous system.^[^ ^3^ ^]^ Dysfunction or loss of pericytes will increase the BBB permeability and contribute to neurodegenerative conditions and diabetic retinopathy.^[^ ^3^, ^4^, ^5^ ^]^ The roles of pericytes in tumors are a lot more complicated. On one hand, recruitment of pericytes to vessels promotes endothelial cell survival and stabilizes newly formed vascular sprouts, whereas diminished pericyte coverage has been associated with retarded tumor growth, highlighting pericyte generation as a prerequisite for tumor angiogenesis.^[^ ^6^, ^7^ ^]^ On the other hand, pericyte coverage is positively correlated to vessel maturation, and higher pericyte coverage often predicts a better prognosis, indicating a tumor‐suppressive role of pericytes.^[^ ^8^, ^9^ ^]^ Interestingly, pericytes in tumors demonstrate strong extravascular functions, which not only promote tumor cell proliferation and resistance against treatment, but also facilitate tumor metastasis.^[^ ^10^, ^11^, ^12^, ^13^ ^]^ Therefore, pericytes are critical vascular components with functional heterogeneity particularly in tumors.

Vasculature in the most common malignant brain tumor glioblastoma (GBM) shares similarities with that in brain tissues. GBM vessels, like normal brain vessels, are surrounded by pericytes along the abluminal surfaces of endothelial cells.^[^ ^14^, ^15^ ^]^ Hence, pericytes in GBM have garnered much attention with regard to their implications in the malignant growth of GBM. Pericytes cover endothelial cells and thus contribute to the blood–tumor barrier that attenuates the efficiency of chemotherapy by tempering entry of most therapeutic drugs.^[^ ^14^, ^15^, ^16^ ^]^ Furthermore, cytokines secreted by pericytes act on GBM cells and augment DNA damage repair to cause resistance against temozolomide treatment.^[^ ^11^ ^]^ Importantly, the proportional composition of pericytes in GBM tissues correlates with accelerated tumor recurrence and worse prognosis.^[^ ^14^ ^]^ In addition to primary brain tumors, pericytes are deeply involved in tumor metastasis to brain. It has been recognized for a long time that there is a positive correlation between pericyte numbers in primary tumors and the tendency of metastasis. Lately, a study showed that pericytes along with endothelial cells support growth of the breast‐to‐brain metastatic tumors.^[^ ^13^ ^]^ These facts underscore the protumor roles of pericytes in GBM and other brain tumors, yet their origins and functional subpopulations remain to be explored.

Our previous studies have suggested that a large proportion of pericytes in GBM are derived from glioma stem cells (GSCs), which could be distinguished from normal pericytes by tumor‐specific genetic alterations.^[^ ^7^ ^]^ Consistently, several studies on patient‐derived orthotopic xenografts suggested that vascular pericytes in GBMs were derived from transdifferentiation of tumor cells.^[^ ^7^, ^11^, ^14^ ^]^ Furthermore, pericytes derived from CD44^+^ lung cancer stem cells had been recently identified as a critical malignant population in metastatic brain tumors.^[^ ^12^ ^]^ Whereas these reports proposed the tumor origination of pericytes in brain tumors, in autonomous intracranial GBMs in genetically engineered mice, lineage tracing and transplantation showed that neither pericytes nor endothelial cells were derived from tumor cells.^[^ ^17^ ^]^ The hypothesis that pericytes in tumors may be of normal origins has been supported by investigations on multiple tumors in extracranial organs. In the Rip1Tag2 mouse pancreatic islet tumor, pericytes were traced back to hematopoietic Sca1^+^ stem cells from bone marrow, and thereby should be normal but not tumor cells.^[^ ^18^ ^]^ In addition, microvasculature in prostate and mammary tumors transplanted in NG2‐null mice had negative staining of the pericyte marker NG2, indicating that tumor pericytes came from host animals.^[^ ^19^ ^]^ Given the critical roles of pericytes in tumor vasculature and metastasis, clarification of the tumor or normal origins of pericytes and the corresponding molecular profiles would be vital for understanding tumor development.

Vessels distribute immune cells throughout the body, and vascular cells such as pericytes that have frequent physical contacts with immune cells may participate in immunoregulation. Pericytes keep the permeability of vessels and thus may function to limit infiltration of immune cells. Indeed, leukocyte trafficking increased in genetically engineered pericyte‐deficient mice, which may help the induction of autoimmune neuroinflammation.^[^ ^20^ ^]^ Alternatively, pericyte may generate an amiable environment for immune cells through secretion of cytokines and other signals. Intravital imaging revealed a spatiotemporal macrophage niche alongside pericytes that actively preserved survival and homeostatic functions of macrophages to attenuate atherosclerosis.^[^ ^21^ ^]^ Likewise, microglia with close spatial association with pericytes were detected in mouse and human brains, whose numbers decreased in Alzheimer's disease.^[^ ^22^ ^]^ Along with the rapid progressions in tumor immunology, there has been a strong interest in the roles of pericyte in the tumor immune microenvironment. Similar to the situations in normal tissues, deficiency of pericytes in mice with disrupted platelet‐derived growth factor B (PDGF‐B) signaling led to increased transmigration of myeloid‐derived suppressor cells in experimentally induced breast tumors probably because of the defective tumor vasculature, suggesting a negative correlation between pericytes and immune cell infiltration.^[^ ^23^ ^]^ By contrast, several studies indicated a positive correlation between pericytes and infiltration of protumor immune cells such as M2 macrophages in pancreatic, nasopharyngeal, and brain cancers.^[^ ^13^, ^24^, ^25^ ^]^ These seemingly controversial phenomena were observed in breast‐to‐brain metastatic cancers, in which the leakage of immune cells was ascribed to the impaired vasculature resultant from pericyte depletion, and the recruitment of protumor immune cells was credited to CD276^+^ subpopulations of vascular endothelial and mural cells.^[^ ^13^ ^]^ Nonetheless, the types of pericytes responsible to the impact on the tumor immune microenvironment are worth further investigation.

The heterogeneity of vascular cells and the importance of the BBB has garnered strong attention, resulting in a few decent studies utilizing single‐cell RNA‐sequencing (scRNA‐seq) to investigate vasculatures in normal and tumor tissues. These studies intended to analyze vascular cells including endothelial cells, pericytes, and smooth muscle cells altogether, whereas pericytes were regarded as affiliates to endothelial cells. As a matter of fact, these studies obtained few PDGFRβ^+^ pericytes, usually tens to hundreds of cells per sample,^[^ ^13^, ^15^, ^26^, ^27^, ^28^, ^29^, ^30^ ^]^ which were insufficient for a comprehensive analysis of pericytes. In normal tissues, pericytes basically function as vascular structural cells and thereby could be studied within the scope of vasculature. However, pericytes display pleiotropic traits in tumor tissues and are worth an in‐depth investigation.

In this study, we sought to clarify the origins of vascular pericytes and investigate the heterogeneous pericyte subpopulations in human primary GBM. Using fluorescence‐activated cell sorting (FACS), we obtained 33 572 vascular pericytes marked by CD146 from 5 GBM specimens, with above 4700 pericytes per sample. Chromosomal copy number variation (CNV) analysis demonstrated the existence of both tumor‐ and normal‐originated pericytes (T‐PCs and N‐PCs) that had similar proportions in general but unbalanced distributions in individual patients. Analyses of the intrinsic features of T‐PCs and N‐PCs indicated that they were disparate populations with distinctive characteristics. Through construction a GBM atlas by integration of in‐house and public scRNA‐seq data, we found that T‐PCs and N‐PCs had strong variations in external communications with vascular endothelial cells and immune cells in GBMs. We further identified heterogenous subpopulations in both T‐PCs and N‐PCs, and investigated the subpopulations with potential immunoregulatory functions. We found that a T‐PC metacluster marked by CD44 expression demonstrated a close association with tumor‐associated macrophages (TAMs) in bioinformatic analyses. The CD44^High^ pericyte populations were observed in not only human GBM samples but also GSC‐derived pericytes in vivo and in vitro. Analysis of the transcriptomes from purified GSC‐derived CD44^High^ pericytes indicated that they were comparable to the CD44^High^ T‐PC in GBM tissues. Most importantly, orthotopic xenografts generated by coimplantation of GSC‐derived CD44^High^ pericytes and GSCs relative to the control tumors showed increased infiltration of M2‐subtype macrophages and accelerated tumor growth. Taken together, our study proved the existence of T‐PCs in GBMs, analyzed the functional heterogeneity of T‐PCs and N‐PCs, and unraveled the immunoregulatory roles of CD44^High^ pericytes. These findings will not only deepen the understanding of GBM development but also inspire new antitumor therapeutic strategies.

## 2 Results

### 2.1 ScRNA‐Seq Identified Pericytes from Tumor and Normal Origins in Human Primary GBM

**Figure 1:**
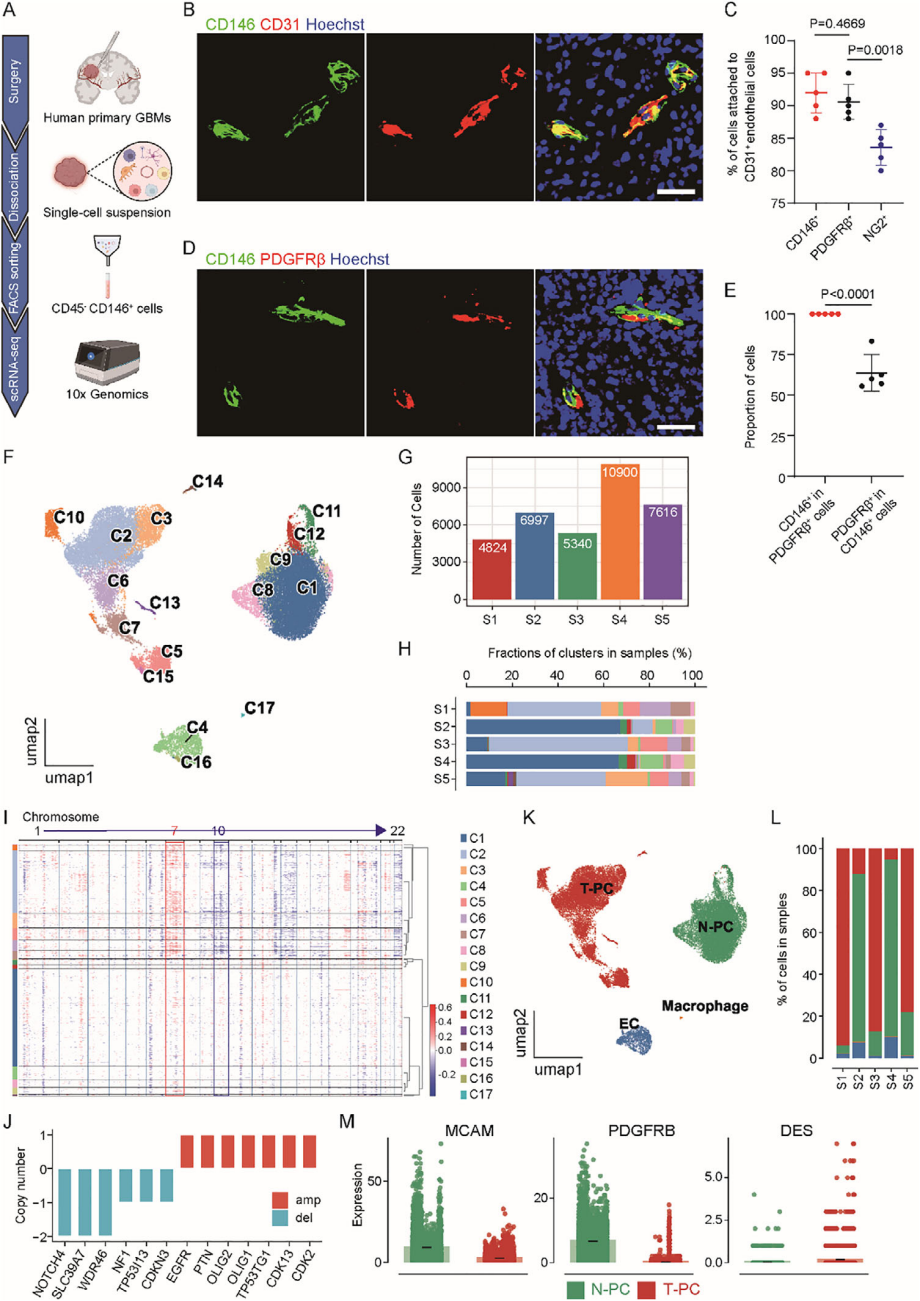
A) scRNA‐seq identified pericytes from tumor and normal origins in human primary GBM. Workflow of the scRNA‐seq analysis of pericytes from human primary GBMs. Fresh surgically resected GBM tumor tissues were immediately dissociated into single‐cell suspensions, which were sorted with FACS to remove CD45^+^ immune cells and collect CD146^+^ pericytes. Purified pericytes were subjected to scRNA‐seq using 10× Genomics Chromium (created with BioRender.com). B) Representative images of immunofluorescent analysis of CD146 (green) and CD31 (red) in human primary GBMs. Frozen sections of human GBMs were immunostained with antibodies against CD146 and CD31, and counterstained with Hoechst to show nuclei (blue). The majority of pericytes attached to the CD31^+^ endothelial cells were positively stained with CD146. Scale bar, 40 µm. C) Statistical quantification of immunofluorescent analysis of the pericyte markers CD146, PDGFRβ, NG2, and the endothelial cell marker CD31 in human primary GBMs. The percentages of pericytes attached to the CD31^+^ endothelial cells that were stained with CD146, PDGFRβ, or NG2 were quantified. Whereas most pericytes were positively stained with CD146 and PDGFRβ, relatively fewer pericytes showed NG2 staining (*n* = 5 sections; mean ± s.d.; two‐tailed unpaired Student's *t*‐test). D) Representative images of immunofluorescent analysis of CD146 (green) and PDGFRβ (red) in human primary GBMs. Frozen sections of human GBMs were immunostained with antibodies against CD146 and PDGFRβ, and counterstained with Hoechst to show nuclei (blue). Almost all PDGFRβ^+^ pericytes had CD146 staining. Yet many CD146^+^ pericytes showed no PDGFRβ signal. Scale bar, 40 µm. E) Statistical quantification of immunofluorescent analysis of the pericyte markers CD146 and PDGFRβ. The percentage of CD146^+^ pericytes with PDGFRβ staining was quantified. Alternatively, the percentage of PDGFRβ^+^ pericytes with CD146 staining was quantified (*n* = 5 sections; mean ± s.d.; two‐tailed unpaired Student's *t*‐test). F) Uniform manifold approximation and projection (UMAP) visualization of the scRNA‐seq data to show the CD146^+^ cell clusters. ScRNA‐seq data were preprocessed and integrated with *Seurat* using classical pipelines, followed by removal of batch effect with the *Harmony* algorithm. All the shown cells (35 677 cells) had CD146 expression. A total of 17 clusters were identified by unsupervised clustering. G) Boxplot showing the numbers of total transcriptomes obtained from individual GBMs. H) Compositions of the 17 identified CD146^+^ cell clusters in individual GBMs. I) Heatmap showing the copy number variation (CNV) in each of the 17 identified CD146^+^ cell clusters using *infercnvpy*. Columns represent the chromosomes in order, and rows denote the CD146^+^ cell clusters automatically grouped. Bar colors represent CNV scores. J) Bar plots showing the indicated genes with amplification or deletion in the CD146^+^ clusters with abnormal CNV in the scRNA‐seq data. Bar colors stand for amplification (red) or deletion (blue). K) UMAP plots showing the indicated types of cells as identified in the scRNA‐seq according to the CNV scores and the differential expression of classical cell type markers of each cell cluster. T‐PC, tumor‐originated pericytes; N‐PC, normal‐originated pericytes; EC, endothelial cells. L) Compositions of T‐PC, N‐PC, EC, and macrophage in individual GBMs. M) Expression levels of the pericyte markers CD146 (MCAM), PDGFRβ (PEGFRB), and Desmin (DES) in T‐PC and N‐PC. Each dot represents a cell in the scRNA‐seq. Abundant CD146 expression was detected in both T‐PC and N‐PC. Yet strong expressions of PDGFRβ and Desmin were detected in N‐PC and T‐PC, respectively.

To take a deep look into pericytes in human primary GBMs, fresh surgically resected tissues from 5 patients were immediately dissociated into single‐cell suspensions. Pericytes were collected with FACS, and immune cells marked by CD45 were excluded in the meantime. ScRNA‐seq of pericytes was carried out using 10× Genomics Chromium (**Figure** **1**A). Multiple surface markers have been reported to label pericytes, and we first sought to find a surface antigen suitable for sorting of pericytes. Immunofluorescent analysis detected abundant CD146^+^ and PDGFRβ^+^ pericytes that predominantly located adjacent to CD31^+^ endothelial cells (Figure 1B,C; Figure S1A–C, Supporting Information). By contrast, relatively fewer pericytes around CD31^+^ endothelial cells were labeled by NG2, whereas many nonpericyte NG2^+^ cells were detected away from vessels (Figure 1C; Figure S1A–C, Supporting Information). Interestingly, the vast majority of PDGFRβ^+^ pericytes were positively stained with CD146^+^, while a few CD146^+^ pericytes were not stained with PDGFRβ (Figure 1D,E). These results suggested that CD146 had the capacity to label most pericytes with strong specificity. Thereafter, we sorted CD45^−^CD146^+^ cells from 5 GBM specimens that accounted for about 2–10% of total cells in individual samples (Figure S1D, Supporting Information), and performed scRNA‐seq. After preprocessing, we obtained 35 677 high‐quality transcriptomes for CD146^+^ cells, ranging from ≈5000 to 10 000 in each patient (Figure 1F,G; Figure S1E,F, Supporting Information). Unsupervised clustering revealed 17 distinct CD146^+^ clusters that were detected in all the 5 patients (Figure 1F,H; Figure S1G, Supporting Information). Most of the clusters showed high expression of multiple pericyte markers and thus were defined as pericytes, which contained a total of 33 572 cells (Figure S1H, Supporting Information). However, the C4 and C16 clusters were characterized as endothelial cells and the C17 cluster was defined as macrophages due to high expression of the respective cell type markers, along with low expression of the pericyte markers (Figure S1H, Supporting Information). These nonpericyte clusters may stand for the CD146^+^ endothelial cells and macrophages that have been reported by several studies.^[^ ^31^, ^32^ ^]^

Clarifying the tumor or normal cell origin of pericytes may not only help the interpretation of the genesis and functions of tumor pericytes but also guide development of vessel‐targeting treatment strategies. There were controversial reports about the origin of pericytes in GBMs, and we investigated this important issue by using our scRNA‐seq data. Macrophages in GBMs are derived from microglia or bone marrow myeloid cells of normal origins. We therefore used macrophages (cluster C17) as the normal reference group, and performed a CNV analysis on all the 17 CD146^+^ clusters. The typical genomic alterations, amplification of chromosome 7 and loss of chromosome 10, were detected in several CD146^+^ clusters (Figure 1I). Consistently, deletion or amplification of several genes involved in tumorigenesis were detected in the CD146^+^ clusters with abnormal CNV (Figure 1J). We assessed the CNV score of each cluster and ranked all the 17 clusters accordingly (Figure S1I, Supporting Information). Thereby, we identified the T‐PCs and the N‐PCs in GBMs (Figure 1K). We further used the *Cancer‐finder* algorithm that identifies malignant cells on the basis of deep learning but not CNV to analyze the 17 CD146^+^ subclusters, and the results confirmed the above discovery of the T‐PC and N‐PC (Figure S1J, Supporting Information). Of note, the C4 and C16 clusters that had been defined as endothelial cells was classified into normal cells in these analyses (Figure S1I,J, Supporting Information), which was consistent with the proposed normal origin of endothelial cells in previous studies.^[^ ^7^ ^]^ Intriguingly, although both T‐PC and N‐PC populations stood for about half of total pericytes in GBMs in general, when it came to individual patient, only one population would take up the majority of pericytes (Figure 1L). Both T‐PCs and N‐PCs had expression of several pericyte markers (Figure S1K, Supporting Information). However, CD146 (MCAM) was vastly expressed in both T‐PCs and N‐PCs, but pericytes with strong PDGFRβ expression were mostly N‐PCs, and most Desmin‐expressing pericytes were T‐PCs (Figure 1M). Some other pericyte markers also showed biased expressions in T‐PC and N‐PC (Figure S1L, Supporting Information), highlighting the distinctiveness of the two pericyte populations. In summary, through scRNA‐seq of CD146^+^ pericytes from human primary GBMs, we clarified that a considerable proportion of pericytes were originated from tumor cells.

### 2.2 T‐PC and N‐PC Are Disparate Populations with Distinctive Characteristics

**Figure 2:**
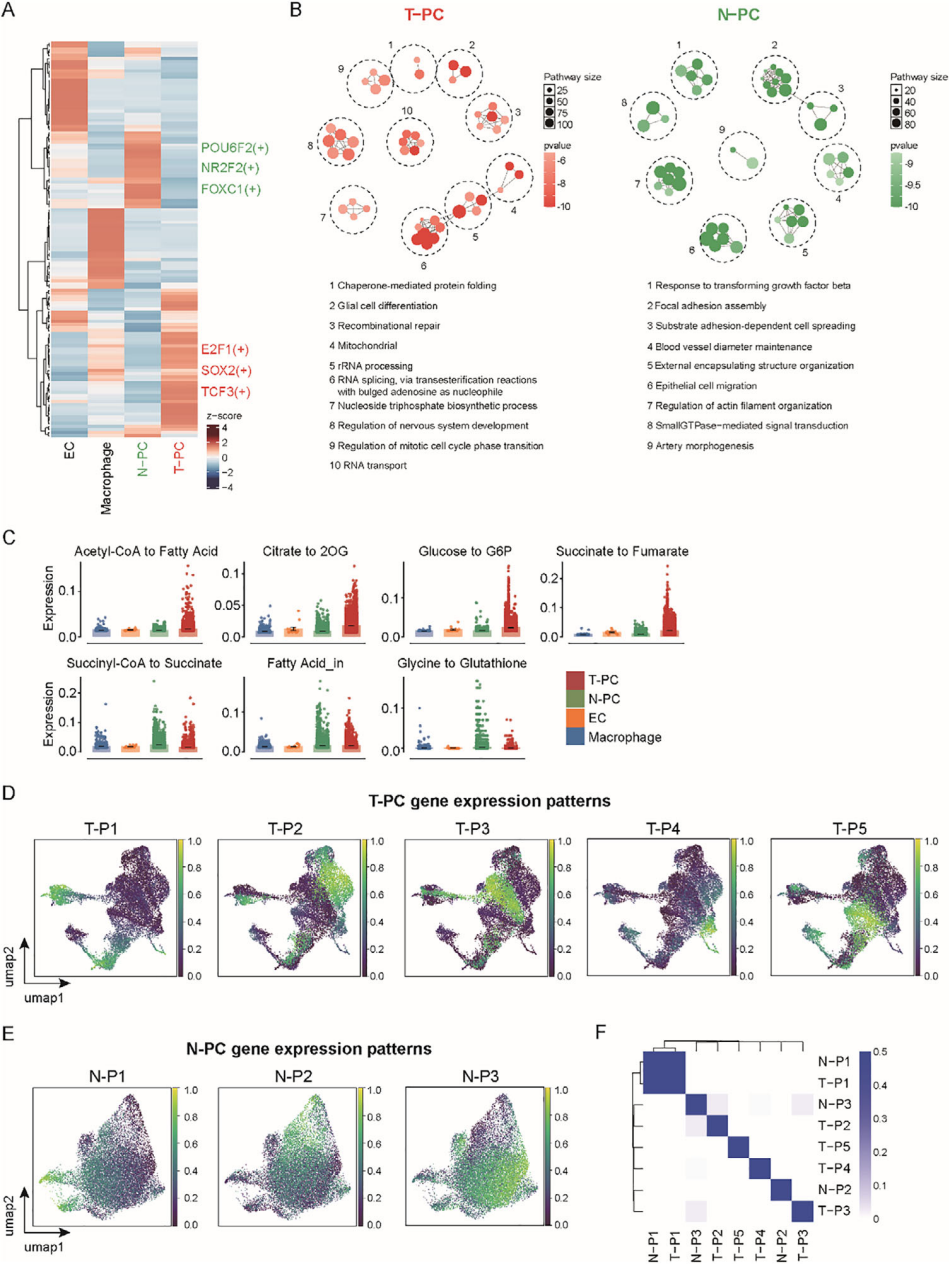
T‐PC and N‐PC are disparate populations with distinctive characteristics. A) Heatmap showing regulon activity scores in T‐PC, N‐PC, EC, and macrophages in the scRNA‐seq data as analyzed by *pySCENIC*. Active regulons in the indicated populations were clustered. Three classic regulons with high activities in T‐PC (in red) and N‐PC (in green), respectively, were shown on the right. Bar colors represent the scaled regulon activity (*z*‐score). All the 4 populations were featured by distinct clusters of regulons. B) Visualization of pathway enrichment networks of N‐PC and T‐PC in the scRNA‐seq data using *aPEAR*. Highly variable genes of N‐PC and T‐PC were inputted for GO enrichment by using *clusterprofiler*. The resultant pathways (dots) were clustered into nodes (dashed circles) representing deduced functions, which were automatically organized into the enrichment networks by the software. Dot color represents *p*‐value and dot size represents the pathway size. T‐PC and N‐PC had distinctive pathway enrichment networks. C) Boxplot showing the metabolic fluxes in T‐PC, N‐PC, EC, and macrophages through analysis of the scRNA‐seq data with *scFEA*. *Y* axis is metabolic flux score. T‐PC and N‐PC showed strong variations in metabolic fluxes. D,E) UMAP plots showing the gene expression patterns of T‐PC (D) and N‐PC (E) at the single‐cell level. The gene expression patterns were determined by analysis of the scRNA‐seq data with *PyCoGAPS*, which defined 5 T‐PC patterns and 3 N‐PC patterns. Color represents the pattern score. The gene expression patterns were generated from all transcriptomes of the scRNA‐seq data, which showed no obvious overlap. F) Heatmap showing the similarity between T‐PC and N‐PC gene expression patterns. Similarity between different patterns were determined by correlation analysis of the genes categorized to gene expression patterns using Jaccard analysis. Visualization was performed with R *pheatmap*. Color intensity represents the correlation level. The T‐P1 T‐PC pattern and the N‐P1 N‐PC pattern had strong similarity. All the other gene expression patterns showed negligible similarity.

Knowing the existence of tumor‐ and normal‐originated pericytes in GBMs, we looked into the characteristics of these two populations. Because transcription factors to a large extent characterize gene transcription in cells, we analyzed the activities of regulons that were defined by transcription factors and their target genes (**Figure** **2**A; Figure S2A, Supporting Information). Not surprisingly, T‐PC and N‐PC were featured by distinctive sets of regulons, with higher activities of SOX2 and E2F1 regulons in the former and NR2F2 and FOXC1 regulons in the latter (Figure 2A; Figure S2A, Supporting Information). Next, we explored the cellular functions of T‐PC and N‐PC by analyzing their intracellular signaling pathways. To this end, the top 200 highly viable genes (HVGs) in T‐PC and N‐PC, respectively, were obtained and inputted for GO enrichment, and the resultant pathways were clustered for pathway enrichment networks (Figure 2B; Figure S2B, Supporting Information). Thereby, we discovered dramatically deviated pathways in T‐PC and N‐PC (Figure 2B), which further indicated the distinctiveness of the two populations. Given that cellular metabolism profoundly influences the status and functions of cells, we evaluated the metabolic features of T‐PC and N‐PC. As expected, remarkable variations in metabolic fluxes were observed between T‐PC and N‐PC (Figure 2C). The above analyses demonstrated a magnificent difference between T‐PC and N‐PC in general. We then asked if T‐PC and N‐PC may have similarities in part. To address this issue, the gene expression patterns of pericytes were analyzed, which revealed 5 patterns in T‐PC and 3 patterns in N‐PC (Figure 2D,E). Similarity between various T‐PC and N‐PC patterns was determined by correlation analysis of the genes belonging to each pattern. The result showed a strong similarity between the T‐P1 and N‐P1 patterns (Figure 2F). Analysis of the genes preferentially expressed in these patterns showed that T‐P1 and N‐P1 both had high expression of TOP2A (Figure S2C,D, Supporting Information), indicating that the two patterns may represent cell proliferation for T‐PC and N‐PC. Except for T‐P1 and N‐P1, all the other gene expression patterns were way different from each other (Figure 2F). Taken together, these data suggest that T‐PC and N‐PC in GBMs are distinctive pericyte populations with little overlap.

### 2.3 T‐PC and N‐PC Have Differential Interactions with the Tumor Microenvironment

**Figure 3:**
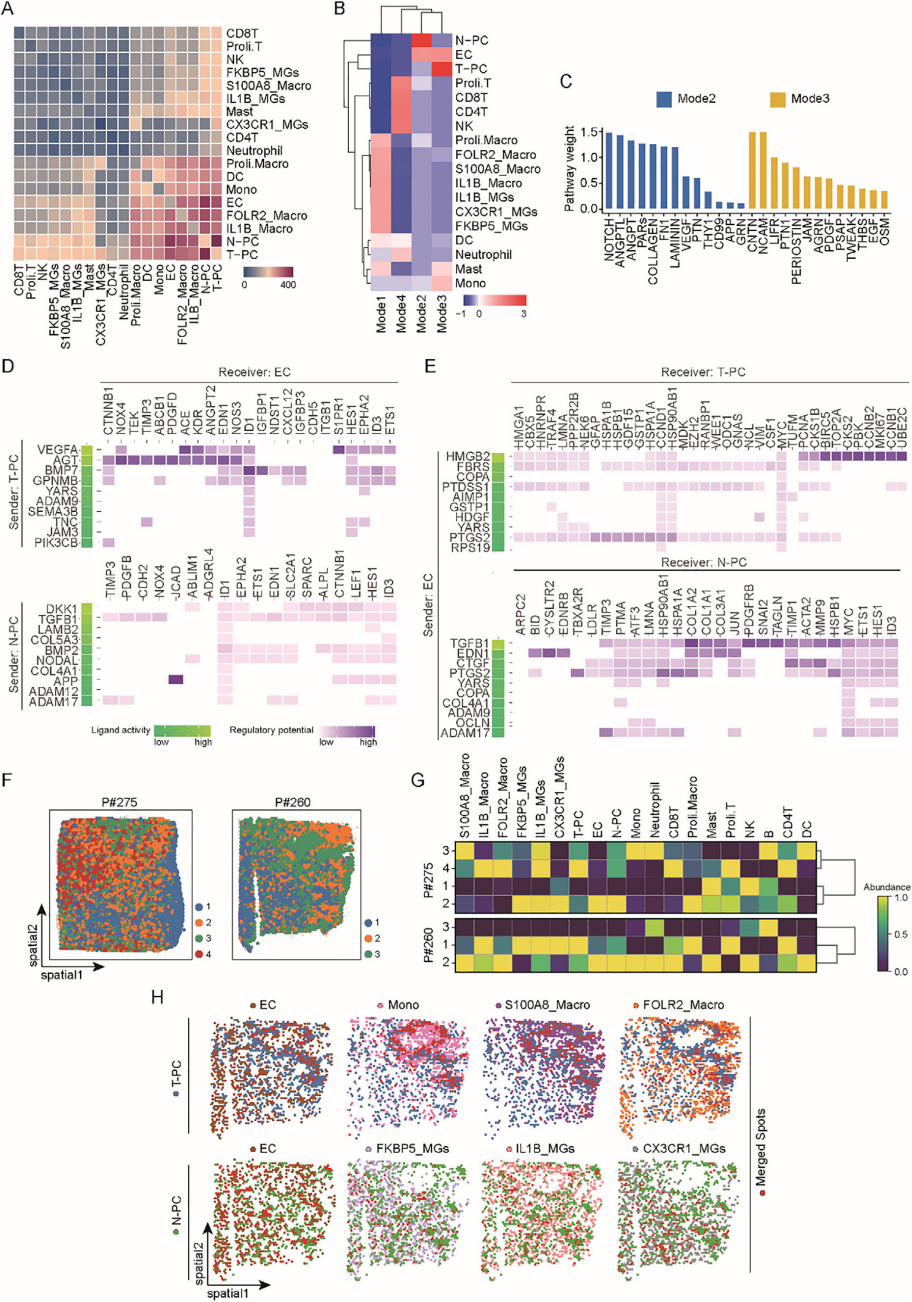
T‐PC and N‐PC have differential interactions with the tumor microenvironment. A) Heatmap showing the strength of intercellular communications between the indicated cell populations according to *CellChat* analysis of ligand–receptor interactions in the GBM atlas derived from integration of in‐house and public scRNA‐seq data. Bar color represents strength of intercellular communication. There were close communications among T‐PC, N‐PC, EC, macrophages, and monocytes. B) Heatmap showing the communication modes comprising the indicated cell populations in the tumor microenvironment according to strength of intercellular communications between cell populations as assessed by *NMF*. Bar color represents mode score. T‐PC and N‐PC were separately assigned to the communication mode2 and mode3, suggesting the difference in their communication with the tumor microenvironment. C) Bar plot showing the top pathways featuring the communication mode2 and mode3. The pathways were determined by using *CellChat* and *NMF*. *Y* axis indicates pathway weight in the communication mode. D,E) Heatmaps showing the signaling axes potentially mediating the influence of pericytes on endothelial cells (D) and vice versa (E) as determined by *NicheNet*. The top10 ligands with the highest expression in the sender cells and the corresponding responsive genes in the receiver cells were shown. Colors represent ligand activities (green) and regulatory potentials (purple). T‐PC and N‐PC applied different sets of signaling axes to communicate with endothelial cells. F) Distributions of the spatial niches discovered in the public spatial transcriptomic data of GBMs. Distribution of cells were analyzed with *Tangram* that integrated the GBM atlas scRNA‐seq and spatial transcriptome. The outputs were clustered to spatial niches by using *Scanpy*. Each sample was divided into multiple niches. G) Heatmap showing the abundance of different cell populations in the indicated spatial niches as discovered in (F). Analysis and visualization were carried out with Python package *scanpy*. Bar color represents the abundance of population. T‐PC and N‐PC were assigned to different niches along with different types of cells. H) Spatial mapping of T‐PC and N‐PC along with the indicated cell populations in the public spatial transcriptomic data of GBMs by using *Tangram*. Spots with high abundance of two cell populations were defined as merged spots. Associated spatial distributions of the indicated cell populations recapitulated their coenrichment in one spatial niche as discovered in (G).

The functions of T‐PC and N‐PC may be inferred in the context of the tumor microenvironment, and thus we investigated the interactions between pericytes and other cell components in tumors. To this end, we obtained public 10× Genomic scRNA‐seq datasets for GBM and normal brain in GEO and reference studies,^[^ ^33^, ^34^, ^35^, ^36^, ^37^, ^38^, ^39^, ^40^ ^]^ and integrated these datasets with our scRNA‐seq data (Figure S3A, Supporting Information). Batch correction and biological conservation were confirmed (Figure S3B, Supporting Information), and the integrated data were further utilized for onward analyses. Based on classical markers and differentially expressed genes (DEGs), unsupervised clustering identified most known cell types. The identified immune cells, endothelial cells, T‐PCs, and N‐PCs from 98 samples were used to construct a single cell atlas of GBM (Figure S3C,D, Supporting Information). To get a finer look into intercellular communications, both macrophages and microglia were further divided into 3 subgroups (Figure S3E, Supporting Information). The intercellular communications between different cell populations in GBM were then assessed which revealed close communications among T‐PC, N‐PC, endothelial cells, macrophages, and monocytes (**Figure** **3**A; Figure S3F, Supporting Information). We then employed the nonnegative matrix factorization (NMF) method to anatomize the above outcome and discovered 4 communication modes (Figure 3B). The types of cells assigned to the same mode should bear similarities in terms of intercellular communication. Interestingly, T‐PC and N‐PC were separately assigned to the communication mode2 and mode3 (Figure 3B) that were featured by distinctive intercellular pathways (Figure 3C). These analyses suggest that T‐PC and N‐PC are active players with different characters in intercellular communications in GBMs.

As two critical components of the vasculature, pericytes and endothelial cells are closely connected physically and functionally. Indeed, we had observed a strong association between endothelial cells and both T‐PC and N‐PC in the analysis of scRNA‐seq data (Figure 3A). We then sought to compare the intercellular connections between endothelial cells and the two pericyte populations, which was achieved using *NicheNet* that linked ligands to target genes in transcriptomics data. Considering that pericytes and endothelial cells mutually regulate each other, we first set pericytes as sender cells and endothelial cells as receiver cells and obtained the top 40 highly expressed ligand genes in pericytes (Figure S3G, Supporting Information). The ligand genes with preferential expression in T‐PC and N‐PC, together with their target genes in endothelial cells, were applied to generate the heatmap to reflect the influence of the two populations on endothelial cells (Figure 3D). The results showed that T‐PC and N‐PC utilized different sets of signaling factors to act on endothelial cells (Figure 3D). Of note, two well‐known proangiogenic factors, vascular endothelial growth factor A (coded by VEGFA) and transforming growth factor beta 1 (coded by TGFB1), stood out as main mediators linking T‐PC and N‐PC with endothelial cells, respectively (Figure 3D). The same approach was utilized to check the influence of endothelial cells as sender cells on T‐PC and N‐PC as receiver cells in communications (Figure 3E). Once again, we found that T‐PC and N‐PC used different sets of genes to respond to endothelial cells (Figure 3E). Therefore, T‐PC and N‐PC both are tightly connected to endothelial cells, yet they apply distinct sets of factors mediating the communications, indicating the difference in their relationships with endothelial cells.

The spatial distribution of pericytes relative to other types of cells in tumor tissues may provide important hints for their connections. We obtained public spatial transcriptomic data and integrated these data with the above GBM atlas. Further analysis divided each GBM section into multiple spatial niches (Figure 3F). The cell populations assigned to the same spatial niche should bear similarities in spatial distributions. Noticeably, in all the samples, T‐PC and N‐PC were located in different spatial niches that contained different sets of immune cells (Figure 3G). Moreover, endothelial cells were often assigned to a spatial niche together with N‐PC rather than T‐PC (Figure 3G). Such spatial distributions were recapitulated through visualization of the cell populations in the GBM sections (Figure 3H). These discoveries suggested that T‐PC and N‐PC had different spatial distributions, which may be associated with their roles in the immune microenvironment and vascular niches.

### 2.4 T‐PC and N‐PC Are Composed of Distinctive Heterogenous Subpopulations

**Figure 4:**
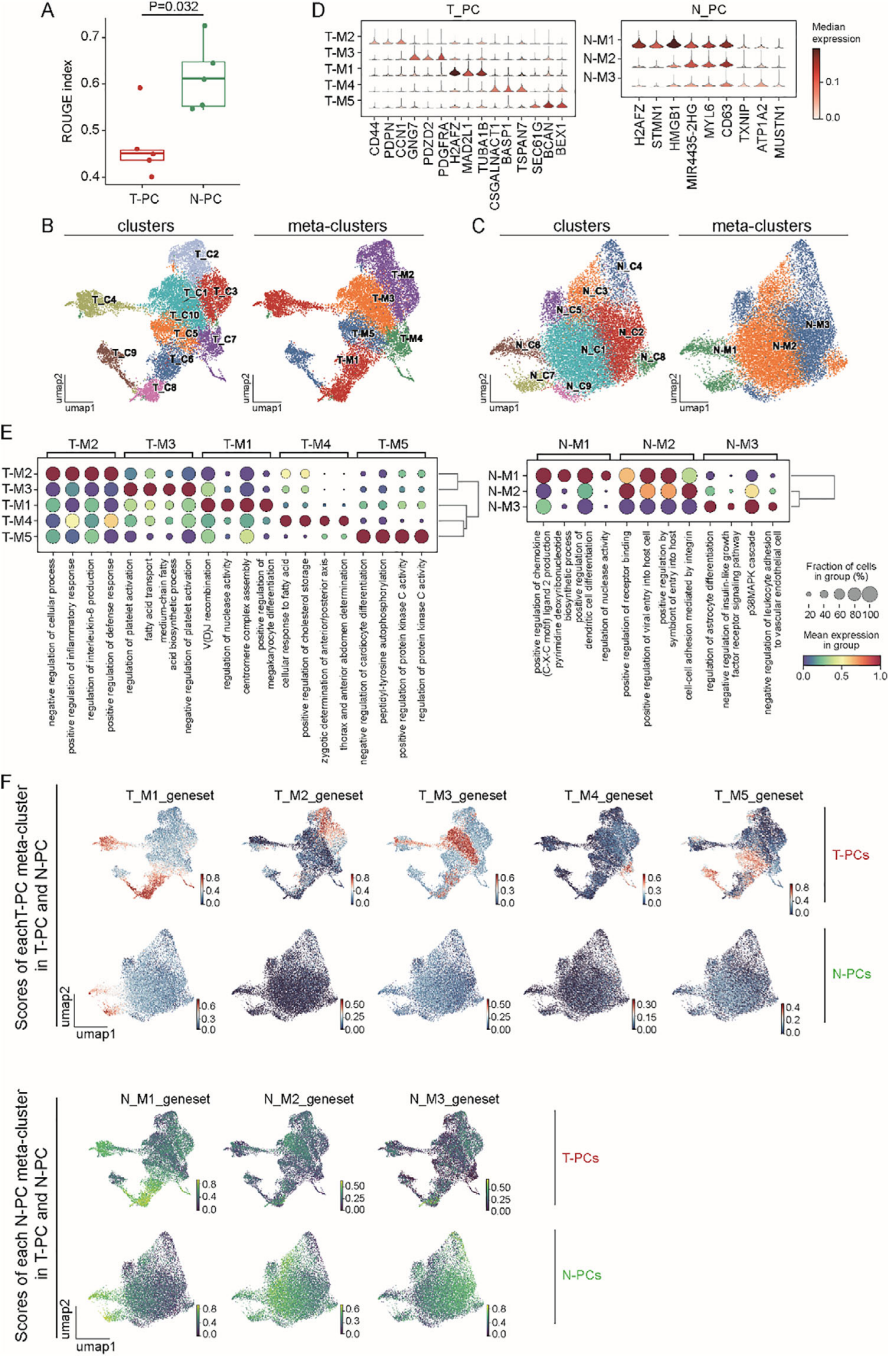
T‐PC and N‐PC are composed of distinctive heterogenous subpopulations. A) Boxplots showing cell purity of T‐PC and N‐PC from the 5 GBM samples through assessment of the scRNA‐seq data with *ROUGE*. Each dot represents a GBM sample. The ROUGE index reflects cell purity. T‐PC in general had lower cell purity relative to N‐PC, suggesting higher heterogeneity in T‐PC (*n* = 5 GBM datasets for T‐PC and N‐PC, *p* = 0.032, Wilcoxon rank‐sum test). B,C) UMAP visualization of the clusters and metaclusters in T‐PC (B) and N‐PC (C). T‐PC and N‐PC identified in the scRNA‐seq data were subjected to unsupervised clustering with *Seurat*, resulting in 10 T‐PC clusters and 9 N‐PC clusters (left panels). Further analysis categorized the T‐PC and N‐PC clusters into 5 and 3 metaclusters, respectively (right panels). D) Violin plot showing the top3 highly variable genes in the T‐PC and N‐PC metaclusters as analyzed by *scanpy*. Color intensity represents the median gene expression. E) Dot plot showing the top4 pathways enriched in the T‐PC and N‐PC metaclusters as analyzed by *AUCell*. Pathway scores were calculated using the scRNA‐seq data based on GO databases. Dot size represents the fraction of metacluster with pathway expression. Dot color represents the scaled expression. The metaclusters were distinguished from each other by unique sets of enriched pathways. F) UMAP visualization of the DEG score of each T‐PC (upper panel) and N‐PC (lower panel) pericyte metacluster in T‐PCs and N‐PCs as assessed by *AUCell*. DEG scores were calculated on the basis of expression levels of DEGs of the metaclusters. Bar color represents expression score of DEGs of the indicated metacluster. In most cases, transcriptomes with high DEG scores of different pericyte metaclusters were separated from each other. Meanwhile, high DEG scores of T‐M1 and N‐M1 metaclusters were observed in the similar part of transcriptomes in the UMAP plot. Therefore, except for T‐M1 and N‐M1, each metacluster was distinguished from the rest metaclusters.

T‐PC and N‐PC may both be composed of heterogenous subpopulations in order to execute diverse functions in the complex tumor microenvironment. We therefore evaluated the cell purity of T‐PC and N‐PC in our scRNA‐seq data with *ROUGE*.^[^ ^41^ ^]^ Interestingly, T‐PC showed a much lower purity in relative to N‐PC (**Figure** **4**A), suggesting a higher heterogeneity of these tumor‐originated cells. To further study the heterogenous subpopulations, T‐PC and N‐PC were reclustered, resulting in 10 and 9 unsupervised clusters in T‐PC and N‐PC, respectively (Figure 4B,C). Based on HVGs of these clusters (Figure S4A, Supporting Information) as well as the gene expression patterns (Figure 2D,E), the T‐PC and N‐PC clusters were further categorized into 5 and 3 metaclusters, respectively (Figure 4B,C). Either the clusters or the metaclusters were detected in every patient (Figure S4B,C, Supporting Information). We next sought to characterize the T‐PC and N‐PC metaclusters. The dramatic difference in the expression of the top3 HVGs in the T‐PC and N‐PC metaclusters indicated the uniqueness of each metacluster (Figure 4D). In addition, the top4 pathways enriched in the T‐PC and N‐PC metaclusters in the scRNA‐seq data were analyzed based on GO databases (Figure 4E). The result showed that each metacluster was distinguished from the rest metaclusters by a unique set of enriched pathways (Figure 4E). Moreover, expression levels of the DEGs of every pericyte metacluster were assessed in each single cell to generate DEG scores (Figure 4F). Uniform manifold approximation and projection (UMAP) visualization revealed distinct separation between transcriptomes with high DEG scores of different pericyte metaclusters. The only exception was that high DEG scores of T‐M1 and N‐M1 metaclusters were observed in the similar part of transcriptomes (Figure 4F). Altogether, these data showed that T‐PC and N‐PC were made of heterogenous metaclusters with unique features.

### 2.5 T‐PC and N‐PC Contain Different Immunoregulatory Subpopulations

**Figure 5:**
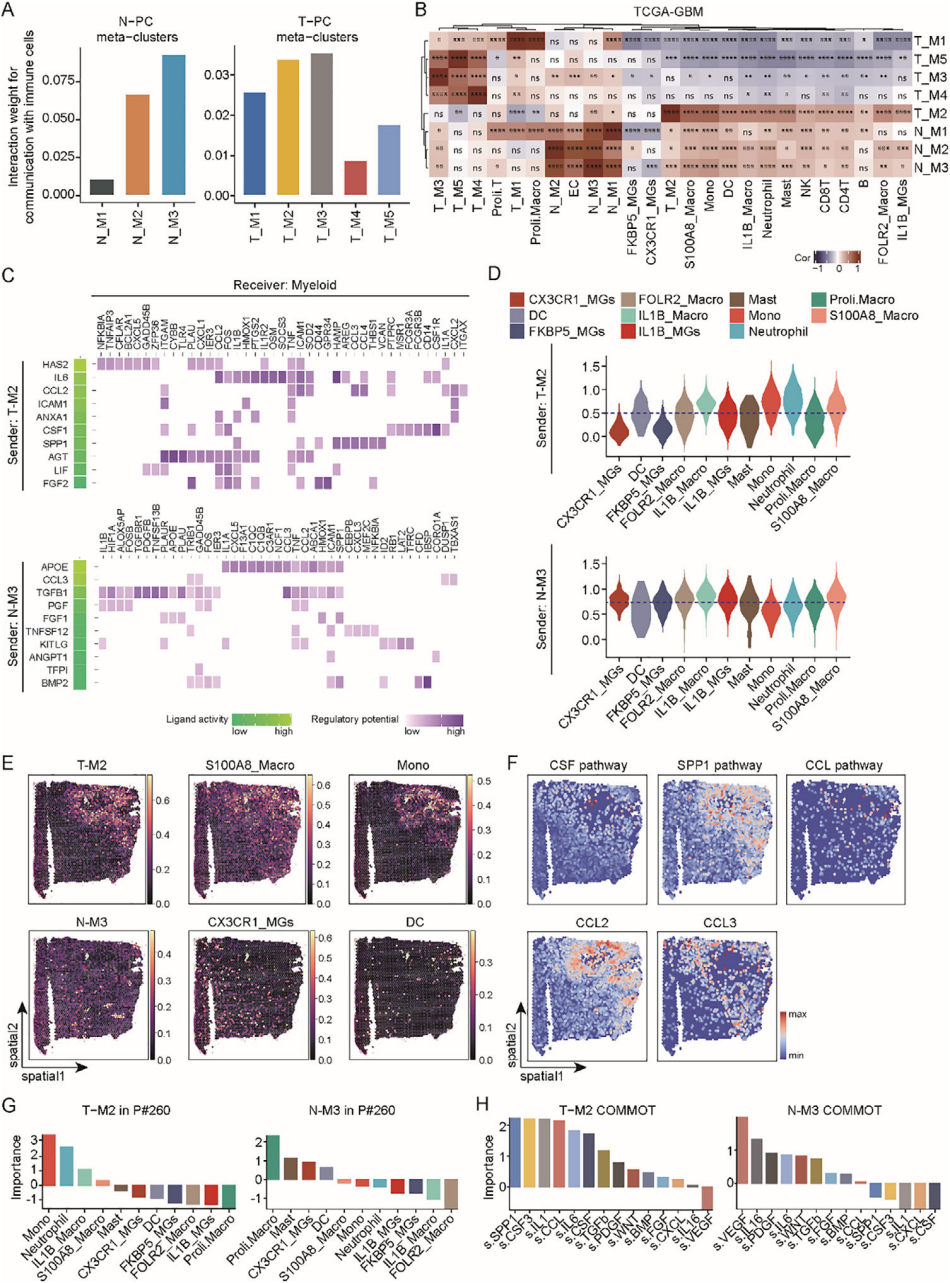
T‐PC and N‐PC contain different immunoregulatory subpopulations. A) Bar plots showing the strength of intercellular communications between the N‐PC (left) and T‐PC (right) metaclusters and immune cells in the GBM atlas derived from integration of in‐house and public scRNA‐seq data. The relative strength of intercellular communications was evaluated using *Cellchat*. B) Heatmap showing Pearson correlations between pericyte metaclusters and the indicated cell populations in GBM patients from TCGA RNA‐seq database. For each patient, the scores of different cell populations were inferred based on the top highly variable genes using *GSVA*. Pearson correlation analysis was performed using the R package *psych*. Color intensity represents Pearson correlation coefficient (Cor). Correlations of statistical significance are marked with asterisks (*, *p* < 0.05; **, *p* < 0.01; ***, *p* < 0.001; ****, *p* < 0.0001). C) Heatmaps showing the signaling axes potentially mediating the influence of the T‐M2 (upper) and N‐M3 (lower) pericyte metaclusters on myeloid cells in the GBM atlas. The top10 ligands with the highest expression in the sender cells and the corresponding responsive genes in the receiver cells were determined by *NicheNet* analysis. Colors represent ligand activities (green) and regulatory potentials (purple). The T‐PC and N‐PC metaclusters applied different sets of signaling axes to influence myeloid cells. D) Violin plots showing the scores of responsive genes in the indicated immune populations in response to ligands from the T‐M2 and N‐M3 pericyte metaclusters in the GBM atlas. Responsive genes were the same as in (C), and the scores were calculated with *Seurat*. Monocytes and S100A8_Macro macrophages tended to react to the T‐M2 T‐PC metacluster, whereas CX3CR1_MG microglia more likely responded to the N‐M3 N‐PC metacluster. E) Spatial mapping of the indicated cell populations in the public spatial transcriptomic data of GBMs by using *Tangram*. Abundances of populations were evaluated through integration of scRNA‐seq and spatial transcriptome. Color represents estimated cell abundance. Cell populations with similar spatial distributions were shown. F) Spatial mapping of the indicated signaling pathways (upper) and genes (lower) in the public spatial transcriptomic data of GBMs by using *COMMOT*. The pathways and genes were from the intercellular signaling axes in (D) that may mediate the influence of T‐M2 or N‐M3 metaclusters on myeloid cells. Color scale represents score of pathway or gene expression level. The pathways and genes showed spatial distributions similar to that of the T‐M2 or N‐M3 as in (E). G) Boxplots showing the levels of codistribution of the T‐M2 or N‐M3 metaclusters with the indicated immune populations in the public spatial transcriptomic data of GBMs. Levels of codistribution were evaluated with *mistyR* on the basis of the abundances and spatial positions of cell populations. Higher colocalization levels stand for more significant spatial correlation. H) Boxplots showing the levels of codistribution of the T‐M2 or N‐M3 metaclusters with the indicated pathways. Levels of codistribution were evaluated with *mistyR* on the basis of the expression of pathways and the abundances and spatial positions of cell populations. Higher colocalization levels stand for more significant spatial correlation.

Our previous analyses had revealed a close association between pericytes and immune cells (Figure 3A). Now that we had categorized T‐PC and N‐PC into metaclusters, we sought to determine the strength of intercellular communications between pericyte metaclusters and immune cells using the GBM atlas generated from integration of in‐house and public 10× Genomic scRNA‐seq data. *Cellchat* analysis of the GBM atlas indicated that the T‐M2 and T‐M3 metaclusters in T‐PC had the strongest interactions with immune cells, and that the N‐M3 was the N‐PC metacluster with the most intensive communications with immune cells (**Figure** **5**A). Moreover, using the gene sets featuring specific cell populations, we scored the pericyte metaclusters and immune cells in individual GBM patients from TCGA database. Correlation analysis of the resultant scores revealed that T‐M2 and N‐M3, relative to other T‐PC and N‐PC metaclusters, showed the most positive associations with immune cells (Figure 5B). Therefore, we focused on the T‐M2 and N‐M3 metaclusters to study the immunoregulatory roles of pericytes.

Myeloid cells took up the majority of immune cells in GBM, and our previous analyses demonstrated an intensive association between pericytes and myeloid cells. We therefore explored the intercellular signaling axes that potentially mediate the influence of the T‐M2 and N‐M3 pericyte metaclusters on myeloid cells in the GBM atlas (Figure S5A,B, Supporting Information). Noticeably, among the top 10 intercellular signal axes, T‐M2 may utilize a set of immunosuppressive factors, including CSF1, SPP1, and *CCL2*, to influence myeloid cells (Figure 5C). By contrast, N‐M3 may apply a bunch of inflammatory factors, such as APOE, *CCL3*, and TGFB1, to act on myeloid cells (Figure 5C). We further scored the responsive genes in the myeloid populations to evaluate their potential responses to the pericyte metaclusters. The results showed that monocytes and S100A8_Macro macrophages tended to react to T‐PC‐M2, whereas CX3CR1_MG and IL1B_MG microglia more likely responded to N‐PC‐M3 (Figure 5D). Taking advantage of public spatial transcriptomic data, we detected the codistributions of the T‐M2 metacluster with monocytes and S100A8_Macro macrophages, as well as the coenrichment of the N‐M3 metacluster and the CXCR1_MG and IL1B_MG microglia (Figure 5E; Figure S5C,D, Supporting Information). Alternatively, when plotted in the spatial transcriptomic data, the pathways and genes of the top10 intercellular signal axes showed spatial distributions similar to the T‐M2 or N‐M3 metaclusters (Figure 5F; Figure S5E,F, Supporting Information). For example, spatial distributions of the SPP1 pathway and the *CCL2* gene mimicked the T‐M2, whereas distribution of the *CCL3* gene resembled the N‐M3 metacluster (Figure 5F). The spatial codistributions of the pericyte metaclusters with the myeloid subpopulations as well as the intercellular signal axes were further quantified, which highlighted a strong spatial correlation between the T‐M2 metacluster and the monocytes and S100A8_Macro macrophages across GBM sections (Figure 5G,H; Figure S5G, Supporting Information). Taken together, these results strongly suggested that T‐PC‐M2 and N‐PC‐M2 may influence different myeloid subpopulations, especially macrophages, through distinctive intercellular signaling.

### 2.6 Pericytes with High CD44 Expression in GBMs Are Closely Associated with Macrophages

**Figure 6:**
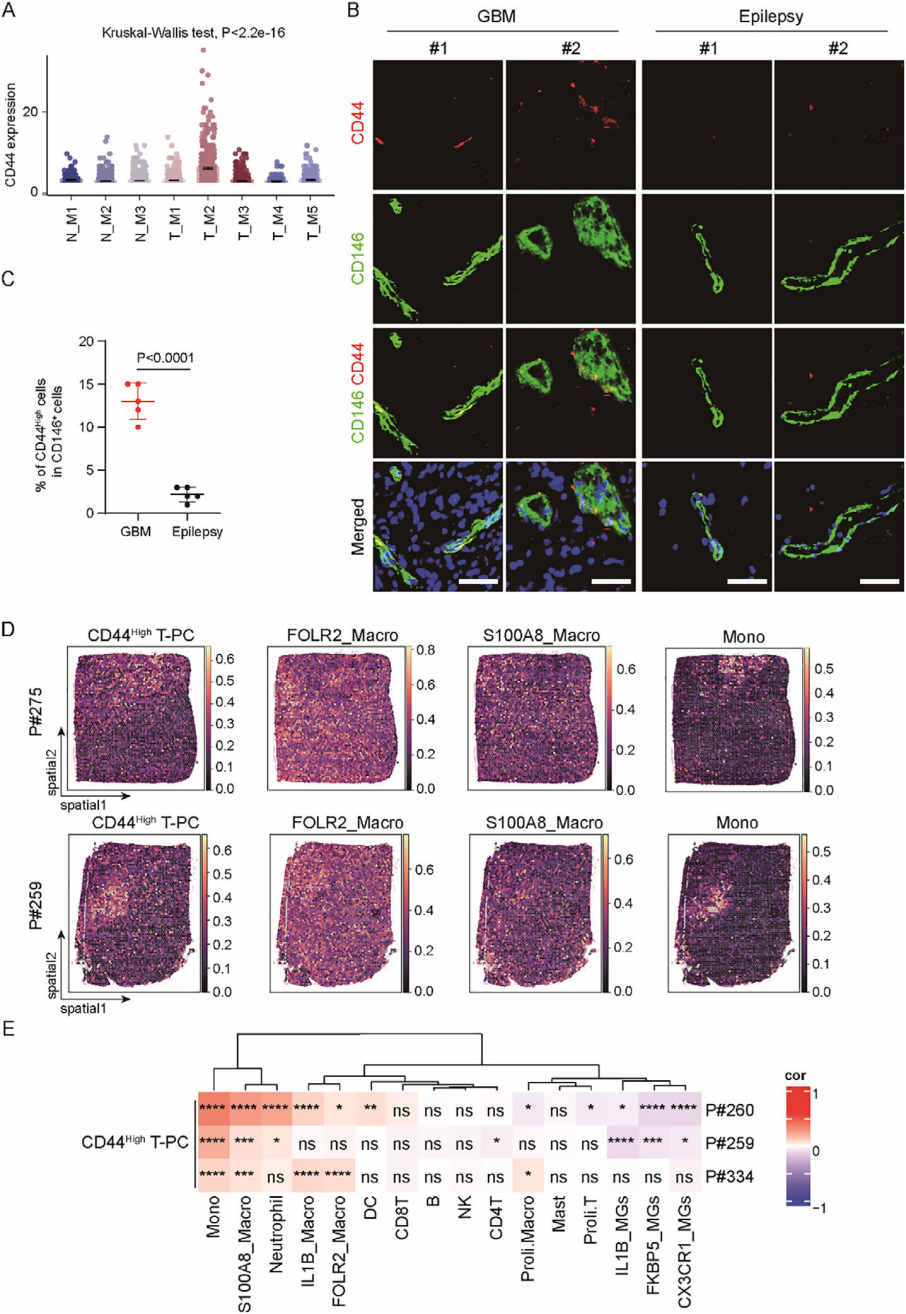
Pericytes with high CD44 expression in GBMs are closely associated with macrophages. A) Boxplot showing the expression of CD44 in pericyte metaclusters in the scRNA‐seq data. Each dot represents a single cell. CD44 expression had dramatic difference between pericyte metaclusters as assessed by Kruskal–Wallis test using *Omicverse*. High expression of CD44 was detected in the T‐M2 T‐PC metacluster rather than other pericyte metaclusters. B,C) Representative images (B) and statistical quantification (C) of immunofluorescent analysis of CD44 (red) and CD146 (green) in tumor tissues from GBM patients and brain tissues from epilepsy patients. Frozen sections were immunostained with antibodies against CD44 and CD146, and counterstained with Hoechst to show nuclei (blue). Part of the CD146^+^ pericytes were positively stained with CD44 in tumor tissues, which was rarely observed in normal brain tissues. Scale bar, 40 µm (*n* = 5 sections for each group; mean ± s.d.; two‐tailed unpaired Student's *t*‐test). D) Spatial mapping of the CD44^High^ T‐PCs, FOLR2_Macro and S100A8_Macro macrophages, and monocytes in the public spatial transcriptomic data of GBMs by using *Tangram*. The CD44^High^ T‐PCs were the collective of T‐PCs with CD44 expression in the scRNA‐seq data that were integrated with the spatial transcriptome. Abundances of populations were evaluated through integration of scRNA‐seq and spatial transcriptome. Color represents estimated cell abundance. These cell populations showed similar spatial distributions. E) Heatmap showing spatial correlations between the CD44^High^ T‐PCs and the indicated immune cell populations in the public spatial transcriptomic data of GBMs. Spatial distributions of cell populations were evaluated with *Tangram* that integrated scRNA‐seq and spatial transcriptome. Pearson correlation analysis was performed using the R package *psych*. Color intensity represents Pearson correlation coefficient (Cor). Correlations of statistical significance are marked with asterisks (*, *p* < 0.05; **, *p* < 0.01; ***, *p* < 0.001; ****, *p* < 0.0001).

On the basis of bioinformatic analysis, we sought to investigate the interaction between T‐PC and macrophages with wet‐lab experiments. We focused on the T‐M2 metacluster and searched for the specific cell surface antigen. Our scRNA‐seq data showed that the T‐M2 rather than other T‐PC or N‐PC megaclusters was featured by CD44 expression (**Figure**s 4D and **6**A). Consistently, immunofluorescent analysis showed that CD146^+^ pericytes in human GBM tissues were partly stained with CD44, whereas pericytes in brain tissues from patients with epilepsy barely had CD44 staining (Figure 6B,C). These results indicated that a proportion of pericytes had high CD44 expression in GBMs but not normal brain tissues. Given that some cells in the T‐M2 metacluster had no CD44 expression, whereas few cells in the other T‐PC metaclusters had CD44 expression (Figure 6A), we regarded all T‐PCs with CD44 expression in the scRNA‐seq data as CD44^High^ T‐PCs to avoid bias and facilitate onward informatic analysis. We then investigated the association between the CD44^High^ T‐PC and macrophages by integration of public spatial transcriptomic data and our scRNA‐seq data. The results showed similar spatial distributions of the CD44^High^ T‐PC, FOLR2_Macro and S100A8_Macro macrophages, and monocytes (Figure 6D). Further correlation analysis showed that the CD44^High^ T‐PCs had a positive correlation with macrophages but a negative correlation with microglia in multiple GBM samples (Figure 6E), suggesting the spatial codistribution of the CD44^High^ T‐PCs and macrophages. The above data demonstrated that a fraction of pericytes with CD44 expression were closely associated with macrophages in GBMs, indicating a potential immunoregulatory role of the CD44^High^ T‐PCs.

### 2.7 GSC‐Derived CD44^High^ Pericytes Resemble the Immunoregulatory CD44^High^ T‐PC

**Figure 7:**
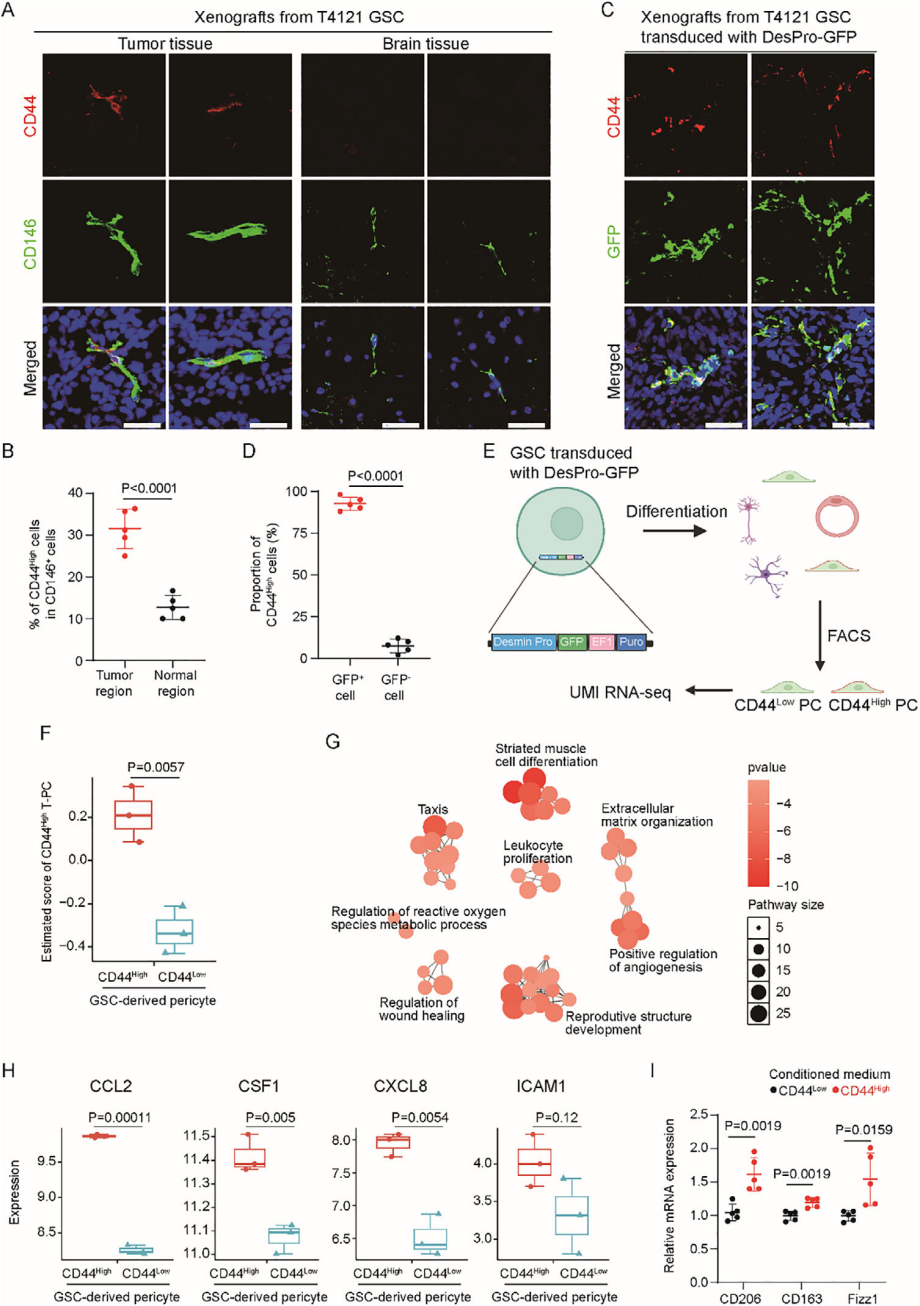
GSC‐derived CD44^High^ pericytes resemble the immunoregulatory CD44^High^ T‐PC. A,B) Representative images (A) and statistical quantification (B) of immunofluorescent analysis of CD44 (red) and CD146 (green) in the tumor and normal brain tissues from mice bearing T4121 GSC‐derived intracranial GBMs. Frozen sections were immunostained with antibodies against CD44 and CD146, and counterstained with Hoechst to show nuclei (blue). Part of the CD146^+^ pericytes were positively stained with CD44 in tumor tissues, which was rarely observed in normal brain tissues. Scale bar, 40 µm (*n* = 5 sections for each group; mean ± s.d.; two‐tailed unpaired Student's *t*‐test). C,D) Representative images (C) and statistical quantification (D) of immunofluorescent staining of CD44 (red) and bioluminescent signals of GFP (green) in the mouse intracranial GBMs derived from T4121 GSC‐transduced with DesPro–GFP. Frozen sections were immunostained with antibodies against CD44, and counterstained with Hoechst to show nuclei (blue). CD44 staining occurred mostly on GSC‐derived GFP^+^ pericytes but rarely on GFP^−^ nonpericyte tumor cells (*n* = 5 sections for each group; mean ± s.d.; two‐tailed unpaired Student's *t*‐test). E) Workflow of the UMI RNA‐seq of GSC‐derived CD44^High^ and CD44^Low^ pericytes. Differentiation of T4121 GSCs transduced with DesPro–GFP were carried out through serum induction for 14 days. Differentiated GBM cells composed of GFP^+^ pericytes and other types of cells were labeled with PE anti‐human CD44 antibodies and subjected to FACS to collect the GFP^+^CD44^High^ and GFP^+^CD44^Low^ pericyte populations, which were used for UMI RNA‐seq (created with BioRender.com). F) Boxplot showing scores of the highly variable genes of CD44^High^ T‐PC in the GSC‐derived CD44^High^ and CD44^Low^ pericytes. For each sample, the scores of CD44^High^ T‐PC HVGs were inferred by *GSVA* analysis of the UMI RNA‐seq data (*n* = 3 replicates for CD44^High^ and CD44^Low^ pericytes; mean ± s.d.; two‐tailed unpaired Student's *t*‐test). G) Visualization of the pathway enrichment network composed of pathways commonly enriched in the GSC‐derived CD44^High^ pericytes and the CD44^High^ T‐PC in human GBMs by using *aPEAR*. DEGs of the GSC‐derived CD44^High^ pericytes and the CD44^High^ T‐PC were obtained from the UMI RNA‐seq and scRNA‐seq data, respectively, which were used as input for GO enrichment using *clusterprofiler*. The commonly enriched pathways were clustered for automatic generation of the enrichment network. Dot color represents *p*‐value and dot size represents the pathway size. H) Boxplot showing expressions of the indicated cytokine‐coding genes in the GSC‐derived CD44^High^ and CD44^Low^ pericytes in UMI RNA‐seq data. These cytokines were highly expressed in the T‐M2 T‐PC metacluster in the scRNA‐seq data. The cytokines had higher expression in the GSC‐derived CD44^High^ relative to CD44^Low^ pericytes, suggesting the comparability of the GSC‐derived CD44^High^ pericytes and the immunoregulatory T‐PC in GBMs (*n* = 3 replicates for CD44^High^ and CD44^Low^ pericytes; mean ± s.d.; two‐tailed unpaired Student's *t*‐test). I) Quantitative PCR analysis of the indicated M2 macrophage markers in THP1 cells cultured in conditioned medium from the GSC‐derived CD44^High^ and CD44^Low^ pericytes. THP1 cells were primed with PMA for 2 days and then cultured in the conditioned medium for 5 days before harvest. Increased expression of M2 markers in THP1 cells were observed when cultured in conditioned medium from the CD44^High^ relative to CD44^Low^ pericytes (*n* = 5 biological repeats; mean ± s.d.; two‐tailed unpaired Student's *t*‐test).

We had previously discovered that the majority of pericytes in tumor tissues were differentiated from GSCs in intracranial GBMs in nude mice.^[^ ^7^, ^14^ ^]^ Now that the CD44^High^ T‐PC in human GBMs may have potential immunoregulatory functions, we checked if GSCs could give rise to CD44^High^ pericytes. Immunofluorescent analysis of GSC‐derived orthotopic GBMs showed that the CD146^+^ pericytes were partly stained with CD44, whereas pericytes in normal brain tissues from tumor‐bearing mice barely had CD44 staining (**Figure** **7**A,B), indicating that a CD44^High^ pericyte subpopulation existed in GBM xenografts but not brain tissues. Our scRNA‐seq data showed that the pericyte marker Desmin had a preferential expression in T‐PC (Figure 1M). Meanwhile, immunofluorescent staining of GSC‐derived intracranial xenografts found that most CD146^+^ pericytes had Desmin expression (Figure S6A,B, Supporting Information). Therefore, we were able to trace the GSC‐derived pericytes in orthotopic GBMs generated from GSCs transduced with the Desmin‐promoter‐driven GFP reporter (DesPro–GFP) according to GFP expression.^[^ ^7^, ^14^ ^]^ Taking advantage of the tracing system, we performed immunofluorescent analysis and found that CD44 staining predominantly occurred in pericytes with GFP signals, suggesting that the CD44^High^ pericytes in intracranial GBMs were mainly derived from GSCs (Figure 7C,D). We then investigated if GSC‐derived CD44^High^ pericytes were comparable to the CD44^High^ T‐PC in GBMs identified in our scRNA‐seq data. To this end, GSCs transduced with DesPro–GFP were differentiated with serum induction, and GFP^+^CD44^High^ and GFP^+^CD44^Low^ cells, representing GSC‐derived CD44^High^ and CD44^Low^ pericytes, were sorted through FACS (Figure S6C, Supporting Information), followed by Unique Molecular Identifiers‐based RNA‐Seq (UMI RNA‐seq) (Figure 7E). The HVGs of the CD44^High^ T‐PC in the scRNA‐seq data to a large extent represented the molecular features of the population. Consistently, in the UMI RNA‐seq data, the GSC‐derived CD44^High^ relative to CD44^Low^ pericytes had a much higher score of the CD44^High^ T‐PC HVGs, indicating the higher similarity between the GSC‐derived CD44^High^ pericytes and the CD44^High^ T‐PC in GBMs (Figure 7F). Furthermore, DEGs in the CD44^High^ T‐PC in the scRNA‐seq data were analyzed to get the pathway enrichment network (Figure S6D, Supporting Information). Likewise, we obtained the DEGs of the GSC‐derived CD44^High^ and CD44^Low^ pericytes (Figure S6E, Supporting Information) and generated the pathway enrichment networks for the two populations (Figure S6F,G, Supporting Information). Cross comparison detected several pathways commonly enriched in the CD44^High^ T‐PCs and the GSC‐derived CD44^High^ pericytes, whereas the CD44^High^ T‐PCs and the GSC‐derived CD44^Low^ pericytes shared few enriched pathways (Figure S6H, Supporting Information). Pathway enrichment network of the commonly enriched pathways in GSC‐derived CD44^High^ pericytes and CD44^High^ T‐PCs highlighted the pathways related to immunoregulation, angiogenesis, extracellular matrix, and other biological processes (Figure 7G), which may be the shared features of these two populations. Moreover, the UMI RNA‐seq revealed the upregulated expression of the immunoregulatory factors *CCL2*, CSF1, CXCL8, and ICAM1 in the GSC‐derived CD44^High^ relative to CD44^Low^ pericytes (Figure 7H), which recapitulated the high expression of these cytokines in the immunoregulatory T‐M2 metacluster (Figure 5C). Finally, THP‐1 macrophages exposed to conditioned medium from GSC‐derived CD44^High^ pericytes exhibited upregulated expression of M2 markers (CD206, CD163, and Fizz1) compared to those treated with medium from GSC‐derived CD44^Low^ pericytes (Figure 7I), suggesting that GSC‐derived CD44^High^ pericytes had the potential to promote M2 polarization of macrophage. In summary, GSC‐derived CD44^High^ pericytes to a large extent are comparable to the immunoregulatory CD44^High^ T‐PC in GBMs.

### 2.8 GSC‐Derived CD44^High^ Pericytes Promote GBM Growth and M2 Polarization of TAMs

**Figure 8:**
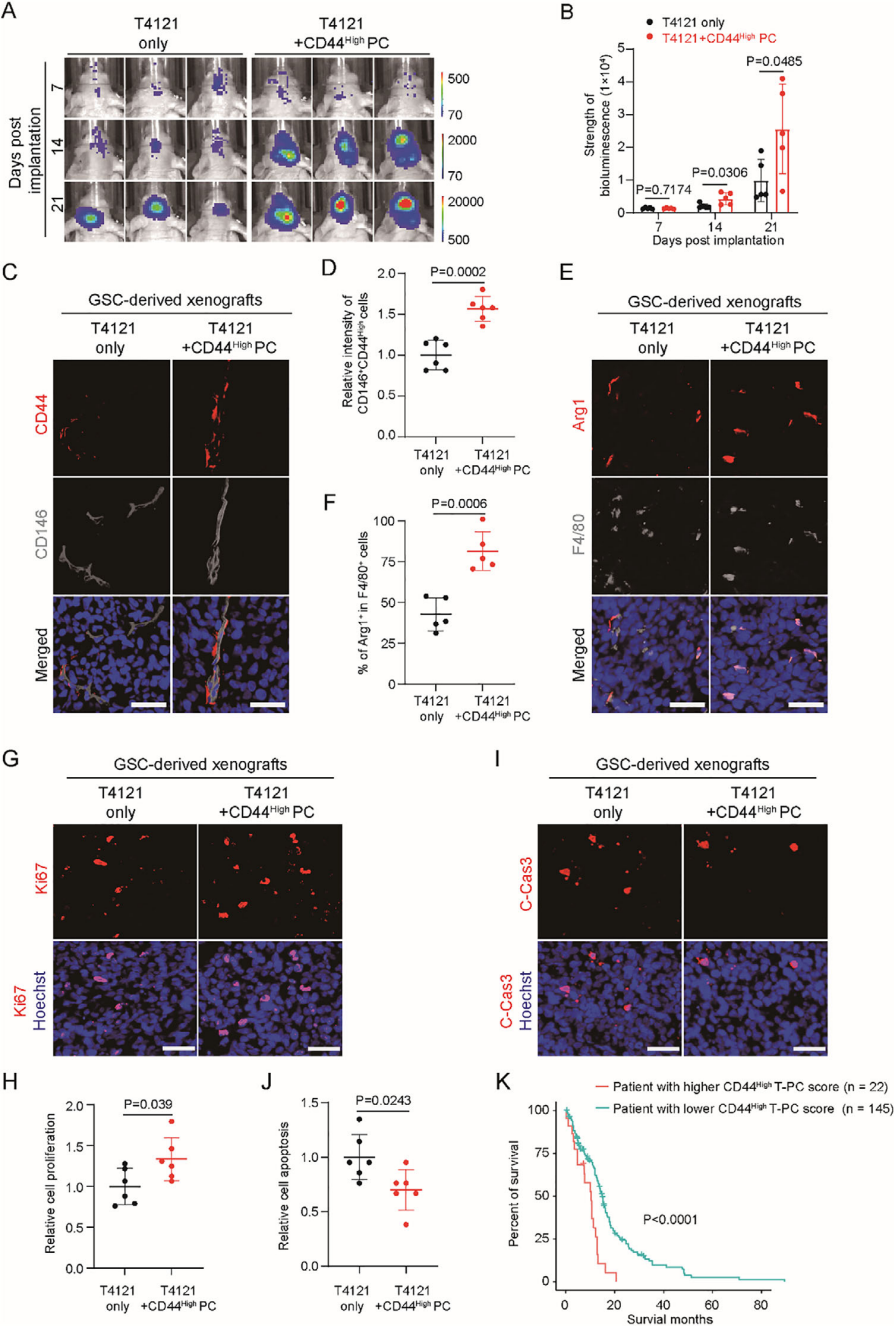
GSC‐derived CD44^High^ pericytes promote GBM growth and M2‐polarization of TAMs. A,B) In vivo bioluminescent images (A) and quantification (B) of intracranial tumor growth in mice bearing GBM xenografts derived from the T4121 GSCs with or without GSC‐derived CD44^High^ pericyte coimplantation. Two‐thousand GSCs transduced with luciferase were implanted into mouse brains with or without 2000 GSC‐derived CD44^High^ pericytes through intracranial injection. Representative bioluminescent images (A) and the strength of bioluminescent signals (B) on day 7, day 14, and day 21 post‐cell‐implantation were shown. Coimplantation of GSC‐derived CD44^High^ pericytes promoted intracranial tumor growth (*n* = 5 mice for each group; mean ± s.d.; two‐tailed unpaired Student's *t*‐test). C,D) Representative images (C) and statistical quantification (D) of immunofluorescent analysis of CD44 (red) and CD146 (gray) in mouse intracranial GBMs derived from the T4121 GSCs with or without GSC‐derived CD44^High^ pericyte coimplantation. Frozen sections were immunostained with antibodies against CD44 and CD146, and counterstained with Hoechst to show nuclei (blue). Xenografts derived from coimplantation of GSC and GSC‐derived CD44^High^ pericytes had more CD146^+^CD44^High^ pericytes relative to those derived from GSC implantation. Scale bar, 40 µm (*n* = 6 sections for each group; mean ± s.d.; two‐tailed unpaired Student's *t*‐test). E,F) Representative images (E) and statistical quantification (F) of immunofluorescent analysis of the M2 macrophage marker Arg1 (red) and the pan‐macrophage marker F4/80 (gray) in mouse intracranial GBMs derived from the T4121 GSCs with or without GSC‐derived CD44^High^ pericyte coimplantation. Frozen sections were immunostained with antibodies against Arg1 and F4/80, and counterstained with Hoechst to show nuclei (blue). Xenografts derived from GSC and GSC‐derived CD44^High^ pericyte coimplantation had higher rates of M2 macrophages relative to those derived from GSC implantation. Scale bar, 40 µm (*n* = 5 sections for each group; mean ± s.d.; two‐tailed unpaired Student's *t*‐test). G,H) Representative images (G) and statistical quantification (H) of immunofluorescent analysis of the cell proliferation marker Ki67 in mouse intracranial GBMs derived from the T4121 GSCs with or without GSC‐derived CD44^High^ pericyte coimplantation. Frozen sections were immunostained with antibodies against Ki67, and counterstained with Hoechst to show nuclei. Xenografts derived from GSC and GSC‐derived CD44^High^ pericyte coimplantation had more active cell proliferation relative to those derived from GSC implantation. Scale bar, 40 µm (*n* = 6 sections for each group; mean ± s.d.; two‐tailed unpaired Student's *t*‐test). I,J) Representative images (I) and statistical quantification (J) of immunofluorescent analysis of the cell apoptosis marker cleaved‐caspase3 in mouse intracranial GBMs derived from the T4121 GSCs with or without GSC‐derived CD44^High^ pericyte coimplantation. Frozen sections were immunostained with antibodies against cleaved‐caspase3, and counterstained with Hoechst to show nuclei. Xenografts derived from GSC and GSC‐derived CD44^High^ pericyte coimplantation had less cell apoptosis relative to those derived from GSC implantation. Scale bar, 40 µm (*n* = 6 sections for each group; mean ± s.d.; two‐tailed unpaired Student's *t*‐test). K) Kaplan–Meier survival analysis of the GBM patients with higher or lower scores of the CD44^High^ T‐PC in the TCGA database. The TCGA RNA‐seq data for GBM patients were scored for the gene signatures of CD44^High^ T‐PC by using *GSVA*. Kaplan–Meier survival curve was generated using *survfit* (*n* = 22 patients for the higher group; *n* = 145 patients for the lower group; two‐tailed log‐rank test).

We next investigated the role of CD44^High^ pericytes in the development of intracranial GBMs in mice. To this end, GFP^+^CD44^High^ pericytes were sorted from cells differentiated from GSCs transduced with DesPro–GFP. GSCs expressing luciferase were implanted into mouse cerebrums either alone or together with GSC‐derived CD44^High^ pericytes. Bioluminescent imaging demonstrated that coimplantation with CD44^High^ pericytes accelerated growth of orthotopic GBMs (**Figure** **8**A,B). Immunofluorescent analysis of the resultant tumor sections with CD44 and CD146 confirmed the increase of CD44^High^ pericytes (Figure 8C,D). To assess the status of TAMs, tumor sections were stained with the M2 macrophage markers Arg1 and CD163, along with the pan macrophage markers F4/80 and Iba1. A higher proportion of M2‐TAMs were detected in xenografts derived from GSC coimplanted with CD44^High^ pericytes relative to the control xenografts (Figure 8E,F; Figure S7A–E, Supporting Information). Meanwhile, the numbers of total TAMs were similar in xenografts derived from GSCs with or without CD44^High^ pericyte coimplantation (Figure S7A, Supporting Information). These data suggested that CD44^High^ pericytes may promote differentiation but not recruitment of TAMs. Because CD44^High^ pericyte coimplantation accelerated tumor growth, we assessed the apoptosis and proliferation of tumor cells. Immunofluorescent staining showed that relative to GSC implantation alone, coimplantation of CD44^High^ pericytes promoted proliferation but inhibited apoptosis of tumor cells (Figure 8G–J). Moreover, immunofluorescent analysis showed that coinjection of CD44^High^ pericytes may promote tumor neovascularization (Figure S7F–H, Supporting Information). Last but not the least, we checked the clinical importance of the CD44^High^ pericytes. GBM patients in the TCGA database were scored based on the gene signatures of CD44^High^ T‐PC. Kaplan–Meier analysis revealed a negative correlation between the score of CD44^High^ T‐PC and overall survival of patients, indicating the protumor role of the CD44^High^ pericytes in GBMs (Figure 8K). Collectively, we found that the GSC‐derived CD44^High^ pericytes had the capacity to promote intracranial GBM growth and M2 polarization of TAMs.

## 3 Discussion

Clarifying the origins of vascular pericytes in GBMs is crucial for understanding tumor angiogenesis and development of targeted therapies, but researchers have not reached an agreement on this issue. Whereas lineage tracing of patient‐derived GSCs in orthotopic mouse xenografts supported the tumor origin of pericytes,^[^ ^7^, ^11^, ^14^ ^]^ studies on autonomous GBMs arising from genetically engineered neural progenitor cells in mice indicated the normal origin of pericytes.^[^ ^17^ ^]^ ScRNA‐seq is a powerful and reliable tool to clarify cell origins, yet most relevant scRNA‐seq studies ignored the issue of pericyte origins probably due to the low numbers of vascular pericytes obtained in these investigations.^[^ ^13^, ^15^, ^26^, ^27^, ^28^, ^29^, ^30^ ^]^ In this study, through scRNA‐seq of a substantial number of CD146^+^ pericytes sorted from human GBM samples, we demonstrated the existence of both T‐PCs and N‐PCs in GBMs. Intriguingly, although T‐PCs and N‐PCs were about half and half in general, there is always a predominant population in individual patient (Figure 1K,L), suggesting a potential mutual exclusion between the two populations. Hence, previous conflicting observations of dominant T‐PCs or N‐PCs in different tumor models may largely be ascribed to their respective experimental conditions that particularly favored pericyte generation from a certain origin. Meanwhile, our data showed that several known pericyte markers had different preferences for T‐PCs and N‐PCs (Figure 1M; Figure S1K,L, Supporting Information). For example, PDGFRβ, the most frequently used pericyte marker in nontumor studies, was vastly expressed in N‐PCs rather than T‐PCs (Figure 1M). Such varied expression patterns of pericyte markers may introduce remarkable bias not only in wet‐lab tracing and detection of pericytes but also in in silico definition of pericytes in scRNA‐seq data from unsorted cells, causing the discrepancies on pericyte origins in various studies. Therefore, our findings to a large extent explain the inconsistences with regard to the origins of vascular pericytes in GBMs.

Isolating pericytes from GBM poses a challenge due to their low abundance in tumor tissues and their strong attachment to endothelial cells. In previous studies, PDGFRβ^+^ pericytes were sorted with either FACS or magnetic‐activated cell sorting (MACS), yet less than 1000 of pericytes were purified from each patient.^[^ ^13^, ^15^, ^30^ ^]^ MACS relative to FACs may help to maintain cell viability, but the processing procedure may cause reaggregation of single cells into clusters that were hard to capture, leading to a low overall recovery of pericytes. Furthermore, without high‐quality antibodies that have both strong affinity and high sensitivity, MACS may not grab pericytes with weak expression of surface antigens. To avoid these problems, we chose FACS to get pericytes for our study. Since immunofluorescent staining detected an exclusive expression of CD146 in pericytes that physically contacted CD31^+^ endothelial cells in GBMs (Figure 1B,C), we obtained CD146^+^ pericytes through FACS sorting for scRNA‐seq analysis. Interestingly, our scRNA‐seq data showed that while both T‐PCs and N‐PCs had high CD146 expression, strong PDGFRβ expression was mainly observed in N‐PCs rather than T‐PCs (Figure 1M; Figure S1K,L, Supporting Information). The weak expression of PDGFRβ in T‐PCs may mitigate the capture of T‐PCs from GBMs in previous studies using PDGFRβ‐based sorting. Anyway, through FACS‐sorting of CD146^+^ pericytes, we got around 5000 pericytes per patient, a number that exceeded any previous reports and was sufficient for a rigorous comprehensive analysis on the origins and heterogeneity of pericytes in GBMs.

Comparison of the T‐PCs and N‐PCs identified in our study showed that they were disparate populations in terms of gene transcriptions, enriched pathways, and metabolic features. Meanwhile, although both T‐PCs and N‐PCs comprised heterogenous subpopulations, T‐PCs seemed to have a higher heterogeneity relative to N‐PCs (Figure 3A). Such difference may result from the generation of T‐PCs and N‐PCs during tumor development. N‐PCs may be the progeny of pericytes preexisting in normal brain vasculature, albeit they may be influenced by tumor cells. Hence, N‐PCs may have a rigid route of cell fate as vascular structure cells. T‐PCs, when arising from tumor cell transdifferentiation, may adopt diverse differentiation states and thus play flexible roles in tumor tissues. Our scRNA‐seq data were derived from sorted pericytes and could not determine the relationship between GSCs and pericytes. Nevertheless, GSCs at the apex of tumor cell differentiation hierarch may have critical contributions to the generation of T‐PCs. In the tumor microenvironment, T‐PCs and N‐PCs as two distinctive populations may play separate roles. Our bioinformatic analyses suggested that T‐PCs had a closer relationship with monocyte‐derived macrophages, whereas N‐PCs were more preferentially associated with microglia, probably indicating their different positions in the regulation of the immune microenvironment. However, further wet‐lab experiments are required to explore the respective roles of T‐PCs and N‐PCs as well as their relationship within tumor tissues.

Our study showed that the CD44^High^ T‐PCs in scRNA‐seq data and the GSC‐derived CD44^High^ pericytes played immunoregulatory roles. In addition, pathway enrichment analysis showed that the CD44^High^ T‐PCs and the GSC‐derived CD44^High^ pericytes were both enriched of the pathways involved in response to extracellular matrix and angiogenesis (Figure 7G), which may reflect the typical functions of pericytes as vessel components. CD44 is a well‐known cell surface adhesion protein whose upregulation was broadly observed in cells with mesenchymal features. Previous studies suggested that CD44 may be a marker of GSCs especially in mesenchymal subtype of GBMs.^[^ ^42^ ^]^ Since differentiation of GSCs should be an important source of T‐PCs in GBMs, the CD44^High^ pericytes may theoretically be the heirs of the CD44^+^ GSCs. However, we obtained GSC‐derived CD44^High^ pericytes that were differentiated from the CD133^+^/CD15^+^ GSCs with 14 days of serum induction, indicating that CD44 expression may be acquired during transdifferentiation. Further studies about the potential multifaceted roles of the CD44^High^ pericytes as well as their progenitors would warrant the therapeutic strategies targeting these malignant pericyte subpopulations for GBM treatment.

Our scRNA‐seq data suggested that not only T‐PCs and N‐PCs but also their subpopulations may be featured by differential expressions of a set of cell surface markers. Furthermore, intercellular interactions between pericytes and other cell components in the tumor microenvironment should be mainly mediated by cytokines and the corresponding receptors. Therefore, multiplex protein quantification at the single‐cell level may be essential for a panoramic comprehension of the ecosystem composed of various pericyte subpopulations as well as immune and vascular cells. One suitable choice may be the antibody‐based multicolor barcode arrays that had been successfully applied to profile secreted proteins, exosomes, and responses of immune cells to tumor cells at the single‐cell level.^[^ ^43^, ^44^, ^45^ ^]^ Moreover, cutting‐edge tools for bioinformatic studies, including machine learning and artificial intelligence, may be utilized to incorporate transcriptomic and proteomic datasets to a multilayered single‐cell atlas. Such efforts would help to consolidate the discoveries of T‐PCs and N‐PCs as distinctive functional pericyte populations. Importantly, the proposed multilayered single‐cell atlas would likely unravel undiscovered signaling networks that may be critical for the dynamic interactions between vascular and immune cells. Nonetheless, the inconsistent cell properties in vitro and in vivo, the limited availability of high‐quality antibodies, and the difficulties in data alignment and standardization across different data types may remain to be technical bottlenecks in the near future.

In summary, our findings show that pericytes in GBMs could come from either tumor‐ or normal origin. The T‐PCs and N‐PCs are disparate types of pericytes, both composed of heterogenous subpopulations with potentially diverse functions. The CD44^High^ T‐PC subset is closely associated with TAMs in GBMs. GSC‐derived CD44^High^ pericytes recapitulate the immunoregulatory CD44^High^ T‐PCs and promote M2‐polarization of TAMs and orthotopic GBM growth in mice. These discoveries would help to deepen the understanding of brain tumor vasculature and inspire the therapeutic strategies targeting tumor vessels and TAMs for GBM treatment.

## 4 Experimental Section

### Ethics Approvals

Deidentified GBM surgical specimens were collected from the First Affiliated Hospital of the University of Science and Technology (Anhui, China) in accordance with an Institutional Research Ethics Committee‐approved protocol. Informed consent was obtained from all subjects and experiments were approved by the Medical Research Ethics Committee of The First Affiliated Hospital of the University of Science and Technology of China. Participants were not compensated for the involvement in this study. All animal protocols were approved by the Animal Research Ethics Committee of the University of Science and Technology of China (USTC), and all animal experiments were performed in accordance to the USTC guidelines for the use of laboratory animals.

### Human GBM Specimens and Glioma‐Derived Cells

GBM tissues were obtained from patients aged 43–75 years. Sex was not considered in the study design because vasculature in GBM was irrelevant to the gender of patients. The 5 GBM samples utilized for scRNA‐seq were from 3 male and 2 female patients, aged from 47 to 75. For immunofluorescent staining, 7 GBM samples from 5 male and 2 female patients, aged from 43 to 73, along with 5 epilepsy samples from 4 male and 1 female patients, aged from 21 to 58, were used. T387, T4121, and H2S GSCs were kind gifts from Dr. Jeremy Rich (University of Pittsburgh). GSCs were isolated from primary GBMs or xenografts and cultured as previously described.^[^ ^46^ ^]^ In brief, tumor cells were isolated from GBM tumors with Papain Dissociation System (Worthington Biochemical, LK003150). After recovery in the stem cell medium (Neurobasal‐A medium (Thermo Fisher, A2477501) supplemented with B27 (Thermo Fisher, 12587010), 10 ng mL^−1^ EGF, 10 ng mL^−1^ bFGF, 2 mm l‐glutamine, and 1 mm sodium pyruvate) in a humidified incubator with 5% CO_2_ for at least 6 h for the reexpression of surface markers, the isolated cells were then subjected to MACS with CD133 microbeads (Miltenyi, 130‐097‐049) and CD15 microbeads (Miltenyi, 130‐046‐601) to obtain the GSC population (CD15^+^/CD133^+^). GSCs were cultured in the stem cell medium in a humidified incubator with 5% CO_2_ for a short period (<5 passages) before further experiments.

### FACS of Pericytes

For purification of pericytes from human GBM samples, fresh surgically resected tissues were immediately dissociated into single‐cell suspensions using Papain Dissociation System (Worthington Biochemical, LK003150). Cells were then resuspended in a 30% Percoll (Santa Cruz, sc‐296039A) phosphate‐buffered saline (PBS) solution and centrifuged at 700*g* for 10 min to remove myelin. After then, cells were incubated with Human TruStain FcX (Biolegend, 422302) to block Fc receptors, followed by incubation with PE antihuman CD146 antibody (Biolegend, 361005) and APC antihuman CD45 antibody (Biolegend, 304012) at 4 °C for 30 min. After then, CD45^−^CD146^+^ pericytes were sorted using flow cytometer (BD, FACSAriaIII). For purification of GSC‐derived CD44^High^ pericytes, GSCs were transduced with DesPro–GFP or DesPro–vector through lentiviral infection. For pericyte differentiation, GSCs were plated at 300 000 cells per dish and maintained in differentiation medium (1640 medium (VivaCell, C3010‐0500) supplemented with 10% fetal bovine serum (FBS) (YEASEN, 40131ES10)) for 14 days, with passaging to the same density on day 8. The differentiated cells were trypsinized into single cell suspensions for FACS. Cells were then incubated with Human TruStain FcX (Biolegend, 422302) to block Fc receptors, followed by incubation with PE antihuman CD44 antibody (Biolegend, 397503) at 4 °C for 30 min. After then, GFP^+^CD44^High^ and GFP^+^CD44^Low^ pericytes were sorted using flow cytometer (BD, FACSAriaIII). Briefly, cells differentiated from GSCs transduced with DesPro–vector were used as negative control, while GFP^+^ and GFP^−^ cells were sorted from differentiated cells transduced with DesPro–GFP. Subsequently, from the GFP^+^ cells, CD44^High^ cells with high PE signals that were apart from the main cell cluster of CD44^Low^ cells with low PE signals in the cytometry scatter plot were sorted and collected as GSC‐derived CD44^High^ and CD44^Low^ pericytes, respectively. Pericyte differentiation and the following FACS analysis were performed with 5 biologically independent repeats. All FACS data analyses were performed using FlowJo_V10 software (BD Biosciences).

### DNA Constructs and Lentivirus Production

DesPro–GFP was constructed by replacing the CMV promoter of the pCDH‐CMV‐MCS‐EF1‐Puro vector (System Biosciences CD510B‐1) with Desmin promoter along with insertion of GFP into the multiple cloning site as described in the previous study.^[^ ^14^ ^]^ For lentivirus production, 293FT cells were transfected with DesPro–GFP plus two helper plasmids pCI‐VSVG and ps‐PAX2. 72 h after transfection, lentiviral supernatant was collected and passed through a 0.45 mm syringe filter. Cells were then infected with lentiviruses and selected with 1 µg mL^−1^ puromycin.

### Immunofluorescent Staining

Immunofluorescent staining was performed as described before.^[^ ^46^ ^]^ Surgical human GBM specimens or intracranial xenografts were fixed overnight in 4% PFA at 4 °C, stored in 30% sucrose solution overnight at 4 °C, embedded in OCT at −20 °C overnight, and cryosectioned at a thickness of 7 µm. Sections were fixed in 4% PFA for 15 min at room temperature. After then, the sections were washed in PBS. Sections were then incubated in a PBS solution containing 5% donkey serum plus 0.3% Triton X‐100 for 1 h at room temperature for blocking and permeabilization. Sections were incubated with primary antibodies (1:200 dilution) in PBS containing 0.3% Triton X‐100 overnight at 4 °C. After then, the sections were washed in PBS and incubated with secondary antibodies (1:1000 dilution) plus Hoechst (1:20 000 dilution) in PBS containing 0.3% Triton X‐100 for 2 h at room temperature in dark. After final wash in PBS, a coverslip was mount on sections, and the staining was subjected to microscopy. Specific antibodies against CD146 (Abcam, ab75769; R&D Systems, AF932), CD31 (Dako, M082301), PDGFRβ (Cell Signaling Technology, 3169S), Desmin (Proteintech, 16520‐1‐AP), NG2 (Miltenyi Biotec, 130‐099‐413), CD44 (Abcam, ab6124; Abcam, ab119348), Ki67 (Abcam, ab15580), cleaved caspase‐3 (Cell Signaling Technology, 9661S), Iba1 (Abcam, ab5076), CD163 (SANTA CRUZ BIOTECHNOLOGY, sc‐20066), F4/80 (Bio‐rad, MCA497GA), Arg1 (SANTA CRUZ BIOTECHNOLOGY, sc‐166920) were used for the staining of GBM tumor sections as indicated. All the primary antibodies were used with a dilution of 1:200.

### Intracranial Tumor Formation

Intracranial transplantation of GSCs to establish orthotopic GBM xenografts was performed as described.^[^ ^14^, ^46^ ^]^ GSCs were infected with luciferase through lentiviral infection. Cells were selected with puromycin (1 µg mL, Fisher Scientific) for 48 h after infection. A total of 2000 GSCs with or without 2000 GSC‐derived CD44^High^ pericytes were then engrafted intracranially into immunocompromised nude mice aged 5–8 weeks (BALB/c nude, SLAC ANIMAL COMPANY) into the right cerebral cortex at a depth of 2.5–3.5 mm. Animals were monitored by bioluminescent imaging and maintained until manifestation of neurological signs. Bioluminescent imaging was used to monitor intracranial GBM growth in mice by intraperitoneal injection of d‐luciferin (150 mg kg^−1^, Goldbio, LUCK‐1G) followed by signal capture with the Spectrum IVIS imaging system (PerkinElmer). No specific method was used to predetermine sample size. The experiments were not randomized. The investigators were not blinded to allocation during experiments and outcome assessment.

### Quantitative Polymerase Chain Reaction (qPCR)

Total RNA was isolated with the EZ‐10 Spin Column Total RNA Isolation Kit (BBI, B610583), reverse transcribed with the PrimeScript RT Master Mix (TAKARA, RR036A), and analyzed by quantitative PCR using SYBR Green PCR Master Mix (Biosharp, BL705A) with the LightCycler 96 real time PCR instrument (Roche). Samples in triplicates were subjected to two‐step real‐time PCR analysis. Cycle threshold (*C*_t_) values were determined automatically by the instrument. The ΔΔCt method was used to calculate relative expression of the target genes by using GAPDH as the internal control. At least five biological repeats were performed for each analysis, whereas a representative result containing three technical replicates were used to generate graphs. Primers sequences were as below. CD206: Forward 5′‐GCACTGGGACTCACTGCAT‐3′; Reverse 5′‐TCCTGGTTTTTGCCTCTGTC‐3′. CD163: Forward: 5′‐GCGGCTTGCAGTTTCCTCAA‐3′; Reverse: 5′‐TGGCTCAGAATGGCCTCCTT‐3′. Fizz1: Forward: 5′‐CACCTCTTCACTCGAGGGACAGTTG‐3′. Reverse: 5′‐GGTCCCAGTGCATATGGATGAGACC‐3′.

### Treatment of THP‐1 Macrophages

For macrophage polarization assays, the primed THP‐1 macrophages were treated with the conditioned medium from GSC‐derived pericytes, followed by qPCR analysis of M2 markers. In detail, sorted GSC‐derived CD44^High^ and CD44^Low^ pericytes were cultured in 1640 medium supplemented with 10% FBS for 5 days and the supernatants were collected as conditioned medium. THP‐1 cells (ATCC, TIB‐202) were initially maintained in 1640 medium supplemented with 10% FBS. To get M0 macrophages, THP‐1 cells were split to 2 million cells per 10 cm dish and primed with PMA (5 nm) for 48 h. For polarization assays, 3 000 000 primed THP‐1 macrophages in a 3.5 cm dish were cultured in the conditioned medium from sorted GSC‐derived CD44^High^ and CD44^Low^ pericytes for 48 h, and total RNA was then harvested for quantitative PCR analysis.

### 10× ScRNA‐Seq Data Generation and Preprocessing

Freshly sorted pericytes were adjusted to 700–1200 cells µL^−1^ in single‐cell suspension according to the 10× Genomics Chromium Next GEM Single Cell 3ʹ Reagent Kits v3.1 (No. 1000268) Operation manual for computer and library construction. The constructed library was sequenced using Illumina Nova 6000 PE150 platform. The CellRanger version (10× Genomics) was used to align reads to the human reference genome (GRCh38), and the raw count matrix for each sample was obtained from the CellRanger UMI matrix output. To get high‐quality datasets, the percentage of counts from mitochondrial RNA and heat shock‐related RNA per cell was calculated first. Cells were then filtered to retain only higher‐quality cells (mitochondrial reads <10%, number of detected genes >200, and number of UMIs >1000). The R package DoubletFinder was applied to estimate potential doublets in each sample.^[^ ^47^ ^]^ The Seurat package was employed for data normalization and scale,^[^ ^48^ ^]^ resulting in identification of 3000 highly variable genes. Principal component analysis (PCA) was then conducted for dimensionality reduction through incorporating the highly variable genes, and the top 30 principal components (PCs) were selected for subsequent analysis. The R package harmony (v.1.0.3) was utilized to mitigate batch effects.^[^ ^49^ ^]^ Unsupervised clustering was conducted with the *FindNeighbors* and *FindClusters* functions, and the identified clusters were visualized using UMAP. DEGs were determined using the *Seurat* and *Scanpy* packages.^[^ ^50^ ^]^ The cell cluster identities were determined by top‐ranked DEGs and previously reported marker genes.

### Copy Number Variation Analysis

The *inferCNV* and *infercnvpy* packages were used to distinguish malignant cells by inferring chromosomal CNVs based on gene expression in the scRNA‐seq data.^[^ ^51^ ^]^ These packages compared the expression intensities of genes across target cells and relate them to the expression in reference cells. Macrophages were set as the reference cells that were used to estimate CNVs for the potential tumor cell populations. A gene ordering file from the human GRCh38 assembly containing each gene's chromosomal start and end positions was created as the input. By calculating the CNV levels for each cell population, populations were classified with significantly higher CNV levels as potential tumor‐originated cell populations. *Cancer‐Finder*, a deep‐learning algorithm, was applied to identify malignant cells in the scRNA‐seq data on python.^[^ ^52^ ^]^ The pretrained model provided by developers was used to infer the malignancy status of cells in the expression matrix of scRNA‐seq data. The threshold was same as default = 0.5. According to the ratio of abnormal cells to normal cells in each population, the populations were ascribed to tumor‐ or normal‐originated cells.

### Gene Regulatory Network (Regulon) Analysis

*PySCENIC* was used to identify gene regulatory networks (regulons) with activity in cell populations.^[^ ^53^, ^54^ ^]^ Briefly, raw count matrix was input, the coexpression network was calculated by *GRNBoost2*, and the regulons were identified by *RcisTarget*. Next, the regulon activity for each cell was scored by *AUCell* based on default settings.^[^ ^53^ ^]^ The acquisition of outputs and processing were performed in R. For each cluster, the average activity of regulons was calculated and ranked.

### Pathway Enrichment Analysis

To identify DEGs in specific clusters, the *FindMarker*s function was employed in *Seurat*. After then, pathway enrichment analyses for T‐PC and N‐PC clusters were performed by *compareCluster* function of the *clusterProfiler* R package with parameter “*q*valueCutoff = 0.01” based on GO BP databases.^[^ ^55^, ^56^ ^]^ The enriched GO signaling pathways were summarized for a better illustration of the functions of T‐PC and N‐PC. These pathways in T‐PC and N‐PC were ranked by *p*‐value, and the top 50 pathways were used to construct pathway enrichment networks using the R package *aPEAR*.^[^ ^57^ ^]^ The clustering method was “hier,” and similarity was “correlation.”

### Estimation of Metabolic Flux

Based on the metabolic heterogeneity between clusters, single‐cell transcriptome expression matrices were used to predict cellular metabolic fluxes in T‐PC and N‐PC using the computational model *scFEA*.^[^ ^58^ ^]^ Human metabolic atlases curated by the developers were utilized to perform the analysis with default parameters, and the resulting data were imported into python for visualization. Metabolic flux of significant difference was determined according to evaluation with the Wilcoxon test.

### Identification of Gene Expression Patterns

Gene expression patterns in T‐PC and N‐PC were assessed using *PyCoGAPS*.^[^ ^59^ ^]^ The T‐PC and N‐PC data were prenormalized using *Scanpy*, and highly variable genes were extracted for onward analysis. As recommended by the developers, the number of patterns in *PyCoGAPS* was initially set to a high value, resulting in a few highly similar patterns. Through refining parameters, a reasonable number of distinctive patterns was obtained and the analysis was reran to achieve more reliable results.

### Construction of GBM Atlas Using In‐House and Public ScRNA‐Seq Data

Transcriptomes in 8 public scRNA‐seq datasets using 10× platform (80 GBM tumor tissues, 8 benign tissues, and 5 normal brain tissues) were obtained. In particular, datasets were from GSE 141552, GSE138794, GSE162631, GSE103224, GSE141383, GSE182109, GSE163108, and GSE173278. After obtaining the preprocessed scRNA‐seq data from GEO, output folders of *cellranger* were loaded in the *Read10X* function of the *Seurat* (v.4.3.0) package, and count matrices were loaded in the *fread* function for each dataset. Subsequently, the *merge* function was applied to integrate sample data into a unified Seurat object along with unique barcode labels. Cells with fewer than 200 detected genes or with more than 20% mitochondrial content out of total detected genes were discarded. The *Seurat* package was employed for data normalization, resulting in identification of 3000 highly variable genes. PCA was then conducted for dimensionality reduction through incorporating the highly variable genes, and the top 30 PCs were selected for subsequent analysis. The R package *harmony* (v.1.0.3) was utilized to mitigate batch effects. Unsupervised clustering was conducted with the *FindNeighbors* and *FindClusters* functions, and the identified clusters were visualized using UMAP. Clusters were identified using the *FindAllMarkers* function of the *Seurat* package and the *COSG_markers* function of the *COSG* (v.0.9.0) package that provided subgroup‐specific genes. The identified subsets were named based on the classical markers and the DEGs. Transcriptomes of immune and vascular populations for each dataset were obtained and then all these cells were integrated with the in‐house scRNA‐seq data. The integrated gene expression matrix was normalized and the top 3000 HVGs were detected. Data were scaled using the *ScaleData* function based on the donor effects and percentages of mitochondrial genes. To remove batch effects, cluster similarity spectrum (CSS) was calculated using the function *cluster_sim_spectrum* in the R package *simspec* with the default parameters and R package *harmony*, respectively.^[^ ^60^ ^]^ UMAP was used to present the outcomes of the CSS and harmony processes. The python package *scib* from *omicverse* was used to evaluate the quality of the integration.^[^ ^61^, ^62^ ^]^

### Analysis of Intercellular Communications

For intercellular interactions between cell populations in the tumor microenvironment, *CellChat* was performed on CellChat object constructed with scRNA‐seq data.^[^ ^63^ ^]^ To better demonstrate the outputs from *CellChat*, the R package *ktplots* was used. The strengths of interactions in the intercellular interaction network were visualized with heatmap. To assess the interactions between cell populations in tumor microenvironment and determine the communication modes, the probability of intercellular interactions was summed from the perspectives of sender and receiver cells. The R package *NMF* was then used to summarize the communication modes.^[^ ^64^ ^]^ The number of NMF modes was determined by checking the decline of the cophenetic curve. Analysis of intercellular communications was also carried out using *CellphonedDB*.^[^ ^65^ ^]^ In that case, raw count matrix extracted from the Seurat object and an annotation file of cell types were used as input files. The *ktplost* package was used to illustrate the frequency of interactions between cell populations. To predict ligands in sender cells with the capacity to induce responsive genes in receiver cells, the *NicheNet* algorithm was used to infer ligand and the corresponding responsive genes by analyzing the expression profiles of specific sender and receiver cells.^[^ ^66^, ^67^, ^68^ ^]^ DEGs with *p*‐values smaller than 0.05 were first obtained by setting endothelial cells as receiver cells and T‐PC and N‐PC as sender cells. The top 40 ligands were then identified and their ligand activity was assessed. Next, the top 10 highly expressed ligands in T‐PC and N‐PC were selected and their corresponding receptors and target genes in endothelial cells (ECs) were identified. The same method was used while setting endothelial cells as sender cells and T‐PC and N‐PC as receiver cells.

### Collection of Spatial Transcriptomics Data

Published spatial transcriptomics datasets for GBM were downloaded from public resources (https://github.com/theMILOlab/SPATAData). Data were integrated and processed by *Seurat*. The spatial data were subjected to normalization using *SCTransform*. Gene expression levels and ratio of mitochondrial genes in every spot (55 µm in diameter, 20–30 cells) were assessed but any spot in the tumor tissues was not filtered to maintain the integrity of the sections.

### Spatial Deconvolution (Tangram)

For the spatial distributions of the cell populations identified by the analysis of the scRNA‐seq data of the GBM atlas, *Tangram* was employed to integrate the scRNA‐seq data with the 10× Genomics Visium mRNA count matrix.^[^ ^69^ ^]^ Briefly, the *Tangram* model backconvolves mRNA counts from 10× Visium data using transcriptional signatures of a reference cell type to estimate the abundance of different cell populations at each spatial spot. This model was implemented through the *Tangram* function in the *omicverse* on python. The GBM atlas was used as the reference single‐cell data that included the cell populations of interest. Highly variable genes that were common between the reference single‐cell data and the spatial transcriptomics data were then identified, which were used for model training. Second, the *cells* model was selected to infer the proportions of various cell types in each spot of the spatial transcriptomics data. For training, the model was run for more than 500 epochs until the stabilization of performance scores. The outputs of Tangram provided the abundance of all cell types in each spatial spot, but lacked proper overviews of tumor sections. Therefore, the outputs using the *sc.pp.neighbors* and *sc.tl.leiden* functions in *Scanpy* were clustered to identify spatial structural domains based on the proportions of cell subpopulations. These structural domains were then merged based on the similarity of the expression abundance of cell populations within each domain, ultimately defining the spatial niche. The colocalization of cell populations was focused upon. For a particular population, the ratio of the population was calculated for each spot, and the top 25% spots with the highest ratio were considered with enrichment of the population. If any pair of cell populations were enriched in one spot, they were defined as colocalized in the spot.

### Identification of Pericyte Metaclusters

Pericyte clusters were identified using the *FindClusters* function. The top 3 DEGs of each subcluster were determined using *Scanpy* with default parameters. Based on the degree of similarity in DEGs, the subclusters were further reclustered into metaclusters.

### TCGA RNA‐Seq Data Analysis

For gene set enrichment analysis, the expression data and clinical information of Genomic Data Commons TCGA were downloaded from UCSC Xena project (http://xena.ucsc.edu). For GBM, samples were scored by using *GSVA*,^[^ ^70^ ^]^ which reflected the relative abundance of indicated cell types. Gene signatures for cell populations were inferred from their DEGs by *COSG*.^[^ ^71^ ^]^ To explore the clinical impact of the CD44^High^ T‐PC, the correlation between the abundance of CD44^High^ T‐PC and the overall survival of GBM patients was determined. Each patient was scored for the abundance of the CD44^High^ T‐PC according to the corresponding gene signature. Patients were then classified into higher and lower groups based on the scores of the CD44^High^ T‐PC. Kaplan–Meier survival curve was generated using *survfit* and plotted using R package *survminer*.^[^ ^72^ ^]^

### Quantification of Spatial Relationship

*COMMOT* was used for screening cell–cell communication in spatial transcriptomics via collective optimal transport on python.^[^ ^73^ ^]^ Spatial transcriptomics data were processed using *Scanpy* as described before. Following the developers' recommendations, the secreted ligand–receptor pairs from the *CellChatDB* ligand–receptor database were utilized for analysis. Ligand–receptor pairs that were expressed in fewer than 2% of the spots were filtered out before further analysis. A spatial distance limit of 500 µm was applied. Finally, *COMMOT* was applied to analyze the direction and intensity of signaling pathway flow, as well as the distribution of internal signaling molecule expression. Multiview Intercellular SpaTial modeling framework was an explainable machine learning framework for knowledge extraction and analysis of spatial transcriptomics data on R.^[^ ^74^ ^]^ The input files included the coordinates of the spots and their Tangram outputs. First, an intraview that captured the cell type proportions within a spot, and a paraview to capture the distribution of cell types in the spots nearby, were defined. The radius was the mean distance to the nearest neighbor plus the standard deviation. And the importance of indicated cell type in predicting distribution of other cell types for each view was plotted separately.

### UMI RNA‐Seq

Total RNA was isolated from the specified biological materials using TRIzol Reagent (Thermo Fisher, 15596026) following the manufacturer's instructions. Samples were then treated with DNase I (NEB, M0303L) to eliminate potential DNA contamination. Purity of RNA was assessed by measuring the A260/A280 ratio using a Nanodrop OneC spectrophotometer (Thermo Fisher). Integrity of RNA was verified using the LabChip GX Touch system (Revvity). Concentration of the qualified RNA was then determined using a Qubit 3.0 fluorometer with the Qubit RNA Broad Range Assay kit (Thermo Fisher, cat. no. Q10210). For library preparation, the total RNA was used as input for RNA sequencing library preparation utilizing the KCTM Digital mRNA Library Prep Kit (Seqhealth Tech. Co., Ltd., Wuhan, China), according to the manufacturer's instructions. The kit eliminated duplication bias and errors in PCR and sequencing steps by using unique molecular identifier of 12 random bases to label the cDNA molecules. The library preparation included the enrichment of PCR products corresponding to fragments ranging from 200 to 500 base pairs. The enriched libraries were quantified and sequenced on a DNBSEQ‐T7 platform (MGI) using the PE150 sequencing mode to generate paired‐end reads.

Normalization and differential analysis between patient groups were conducted with the *DESeq2* R package.^[^ ^75^ ^]^ Differentially expressed genes with *p* < 0.05 and log2(fold change) > 0.25 (case vs control) were used as input for enrichment analysis based on GO BP databases. And visualization was based on *aPEAR*.

### Statistical Analysis

For data statistics of wet‐lab experiments, when comparisons were carried out between two groups, the significance of the difference between two groups was assessed using two‐tailed unpaired Student's *t*‐test. Data were presented as mean ± standard deviation (s.d.), and *p* < 0.05 was considered statistically significant. All these experiments had more than 3 biological replicates. These analyses were performed using GraphPad Prism version 8.0.2. The significance of the Kaplan–Meier curves was assessed using a log‐rank test comparing the different patient or mouse groups. Experimental sample numbers (*n*) and *p*‐values were shown in the figures, and figure legends sections. For transcriptomics data, preprocessing was performed using software as described in method and figure legends sections. Differential analysis was performed and visualized using various bioinformatic tools as described in method and figure legends sections. Data statistics of multiple comparisons were conducted using the built‐in functions with default multiple testing corrections for *p*‐value adjustment. Detailed parameters could be found in user manuals of these bioinformatic tools.

## Conflict of Interest

The authors declare no conflict of interest.

## Author Contributions

C.C. and F.L. contributed equally to this work. H.b.W., A.Z., and W.Z. developed the working hypothesis and scientific concept. C.C., F.L., and W.Z. designed the experiments, analyzed the data, and prepared the manuscript. C.C. and F.L. performed the experiments and organized the data. D.L. provided surgical specimens. H.Y. and H.W. provided pathological reviews. All authors assisted the experiments, helped manuscript preparation, and provided scientific input.

## Supporting information



Supporting Information

## Data Availability

scRNA‐seq datasets have been deposited at the Genome Sequence Archive at the National Genomics Data Center (Beijing, China) that are accessible under BioProject PRJCA025812. UMI RNA‐seq datasets have been deposited at the Genome Sequence Archive at the National Genomics Data Center (Beijing, China) that are accessible under BioProject PRJCA035005. This paper does not report original code.
