# Supplementary material for: Immunoregulatory Roles of Tumor‐Originated Pericytes Identified by Single‐Cell Analysis in Glioblastoma
Source: Adv Sci (Weinh). 2025 Sep 26;12(47):e11856. doi: 10.1002/advs.202511856 (PMC12713092; doi:10.1002/advs.202511856)
Supplement: Supplementary file 1 — Supporting Information [file ADVS-12-e11856-s001.docx]

Supplementary Materials for

**Immunoregulatory roles of tumor-originated pericytes identified by single-cell analysis in glioblastoma**

Cuiying Chu *et al.*

*Wenchao Zhou. Email: [wzaz@ustc.edu.cn](mailto:wzaz@ustc.edu.cn)

**This PDF file includes:**

Figs. S1 to S7

Fig. S1.

**
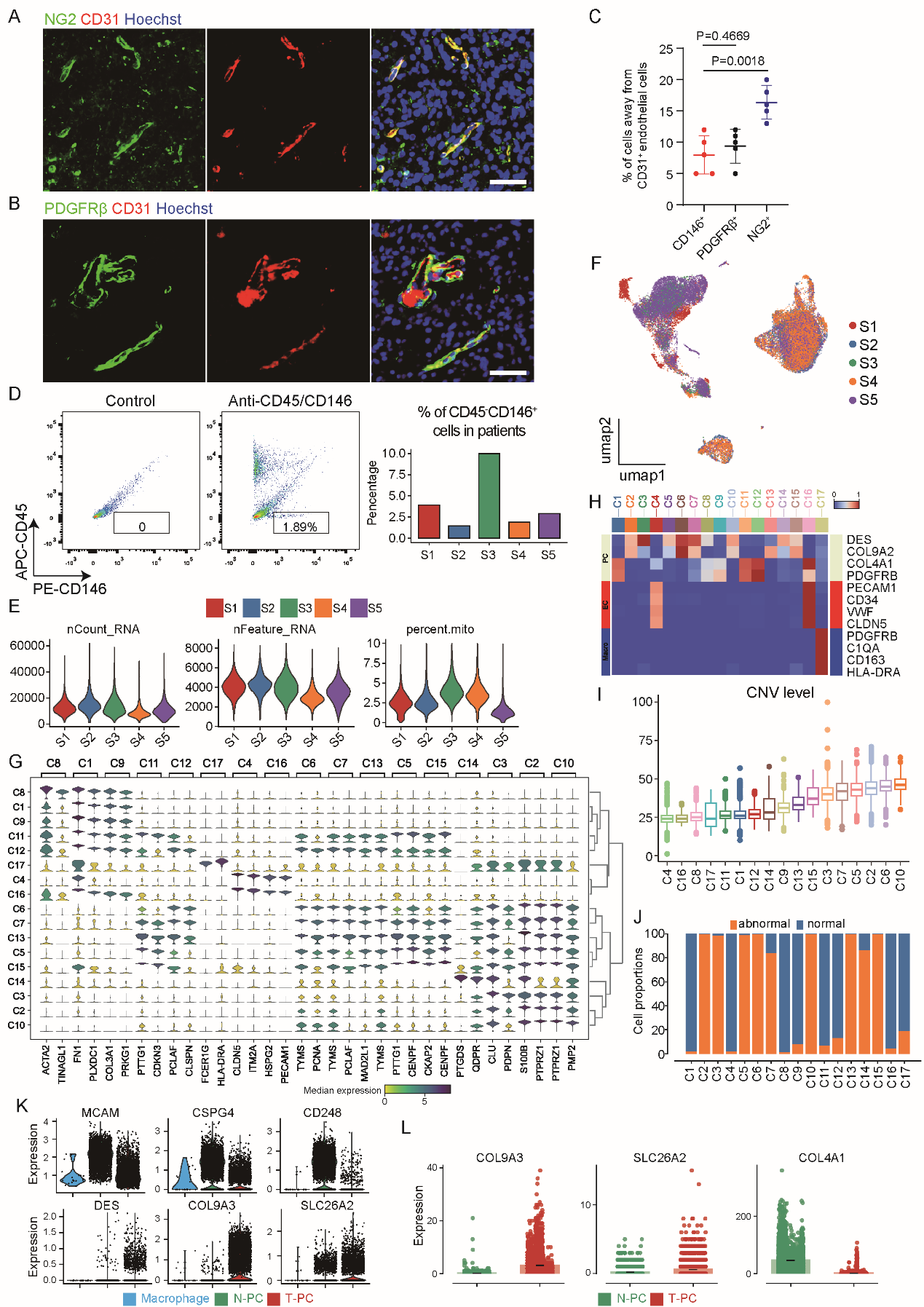
**

**Supplementary Fig S1. scRNA-seq identified pericytes from tumor and normal origins in human primary GBM**

(**A** and **B**) Representative images of immunofluorescent analysis of the pericyte markers NG2 (**A**) or PDGFRβ (**B**) (green) and the endothelial cell marker CD31 (red) in human primary GBMs. Frozen sections of human GBMs were immunostained with antibodies against NG2 or PDGFRβ along with the antibody against CD31, and counterstained with Hoechst to show nuclei (blue). Scale bar, 40 μm.

(**C**) Statistical quantification of immunofluorescent analysis of CD146, PDGFRβ, NG2, and the endothelial cell marker CD31 in human primary GBMs. The percentages of the cells marked by CD146, PDGFRβ, or NG2 that were away from the CD31^+^ endothelial cells were quantified. CD146^+^ or PDGFRβ^+^ cells were mostly attached to CD31^+^ cells, whereas a few NG2^+^ cells were away from CD31^+^ cells. The cells away from the CD31^+^ endothelial cells were considered as non-pericyte cells. (n = 5 sections; mean ± s.d.; two tailed unpaired student’s t-test)

(**D**) Representative flow cytometry plots of CD45^-^CD146^+^ cells in human primary GBM samples. Fresh surgically resected tumor tissues were dissociated into single cell suspensions and labeled with APC anti-human CD45 and PE anti-human CD146 antibodies followed by flow cytometry analysis. Control stands for no labelling. Percentages of CD45^-^CD146^+^ cells in 10,000 dissociated cells were quantified.

(**E**) Quality control analysis of the transcriptomes obtained by scRNA-seq. The number of genes detected in each cell (nFeature_RNA) greater than 200, the total number of molecules detected within a cell (nCount_RNA) greater than 1,000, and percentage of mitochondria genes (percent.mito) lower than 10% were set as the threshold.

(**F**) UMAP plots showing the patient origins of transcriptomes in the scRNA-seq data. Different colors represent different patients.

(**G**) Violin plots showing the expression levels of the top 2 highly variable genes for each cell cluster, with top marker genes highlighted. Bar colors represent the normalized gene expression.

(**H**) Heatmap showing the differential expression of the indicated cell type markers in the 17 cell clusters in the scRNA-seq data. Genes coding for pericyte markers PDGFRA, PDGFRB, DES, COL9A2, and COL4A1, endothelial cell markers PECAM1 (CD31), CD34, CLDN5, and VWF, and macrophage markers AIF1 (Iba1), C1QA, HLA-DRA, and CD163 were included. Bar colors represent the normalized gene expression.

(**I**) Copy number variation (CNV) score of each cluster in the scRNA-seq data using *infercnv*. Cell clusters were displayed according to their CNV scores from low to high.

(**J**) Proportions of cells with normal or abnormal genomes in each of the 17 cell clusters in the scRNA-seq data according to analysis with *Cancer-finder*.

(**K**) Violin plots showing expression levels of the indicated pericyte markers in T-PC and N-PC relative to macrophages in the in-house scRNA-seq data with Seurat. Each dot represents a cell in the scRNA-seq data

(**L**) Expression levels of the pericyte markers COL9A3, SLC26A2, and COL4A1 in T-PC and N-PC. Each dot represents a cell in the scRNA-seq.

Fig. S2.


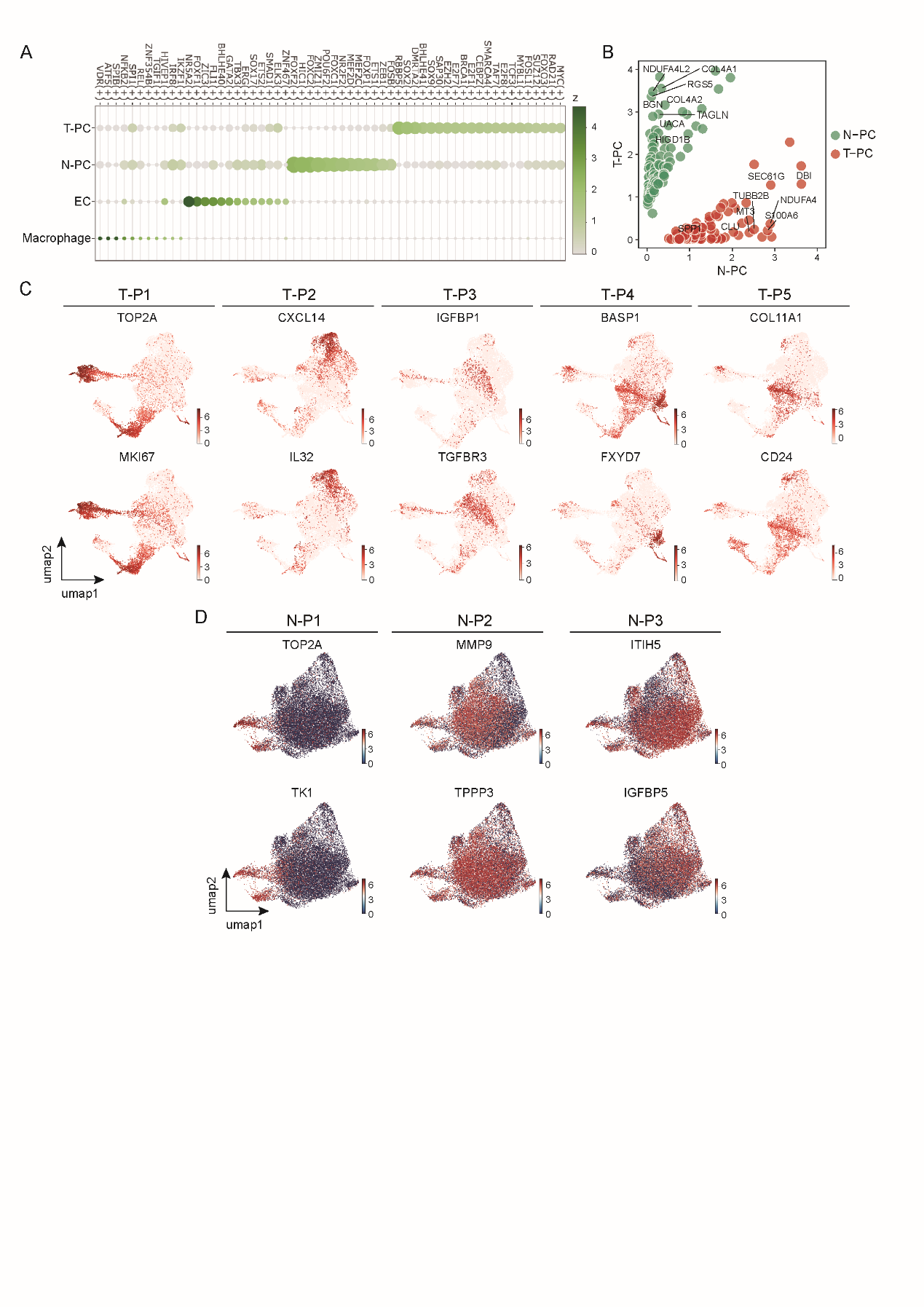


**Supplementary Fig S2. T-PC and N-PC are disparate populations with distinctive characteristics**

(**A**) Dot plot showing all active regulons in T-PC, N-PC, EC, and macrophages in the scRNA-seq data as analyzed by *pySCENIC*. Bar colors represent the scaled regulon activity (z-score).

(**B**) Dot plot showing the highly variable genes in T-PC and N-PC in the scRNA-seq data obtained with *COSG*. The top8 highly variable genes were labeled.

(**C** and **D**) UMAP plots showing expression of the indicated genes featuring different gene expression patterns in T-PC (**C**) and N-PC (**D**). Bar color represents gene expression level.

Fig. S3.


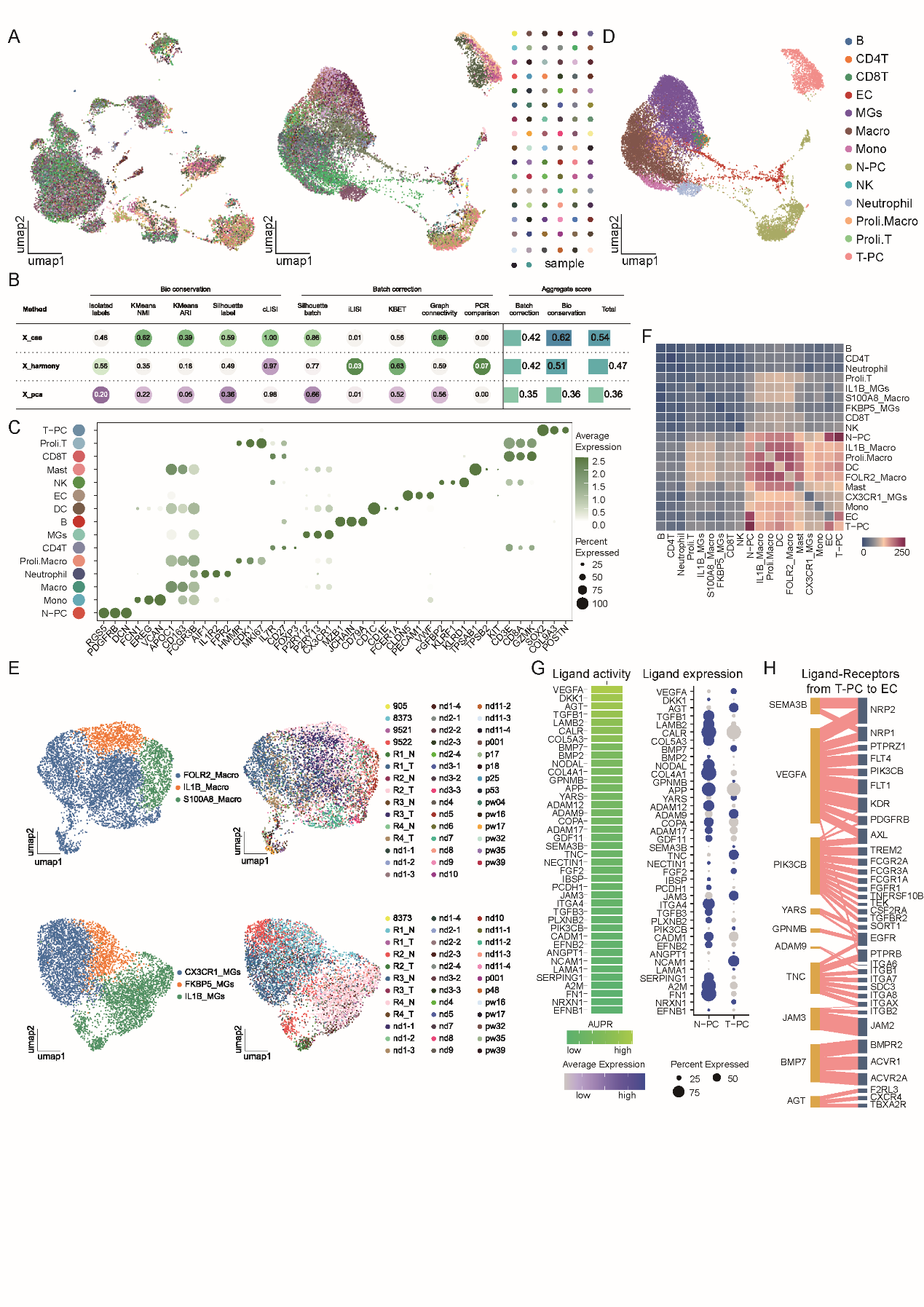


**Supplementary Fig S3. T-PC and N-PC have differential interactions with the tumor microenvironment**

(**A**) UMAP plots showing the patient origin of each transcriptome in the scRNA-seq data generated by integration of in-house and public 10× Genomics datasets. Transcriptomes from public 10× Genomics scRNA-Seq datasets (80 GBM tumor tissues, 8 benign tissues, and 5 normal brain tissues) were preprocessed and integrated with transcriptomes of the CD146^+^ cells in our in-house scRNA-seq data (5 GBM tumor tissues) using *Seurat* following classical pipelines. Batch effect was removed using either the *Harmony* (left) or the *css* (right) algorithm. Different colors represent different patients. A total of 98 samples were included.

(**B**) Evaluation of the outcome of batch effect removal with the indicated algorithms by using the python package *scib*. Processing with *css* demonstrated a better batch correction and biological conservation, and the processed data was used for onward analyses. PCA stands for no batch effect removal.

(**C**) Dot plot showing the top3 highly variable genes of the indicated cell populations in the GBM atlas derived from the integration of in-house and public 10× Genomics datasets. Each cell population was marked by three genes with high expression specifically in the population. Dot size represents the fraction of population expressing the gene. Color intensity represents the scaled gene expression.

(**D**) UMAP plots showing the indicated cell populations as defined in (**C**).

(**E**) UMAP plots showing microglia and macrophage subpopulations in the GBM atlas derived from the integration of in-house and public 10× Genomics datasets. Microglia and macrophage cells defined in (**C**) were subjected to further unsupervised clustering. The resultant subpopulations named by a highly expressed gene were plotted in the left panel. Patient origin of each transcriptome was plotted in the right panel. Dot colors represent subpopulations (left) or patient origins (right).

(**F**) Heatmap showing the strength of intercellular communications between the indicated cell populations according to *Cellphonedb* analysis of ligand-receptor interactions in the GBM atlas derived from the integration of in-house and public 10× Genomics datasets. Bar color represents strength of intercellular communications. There were close communications among T-PC, N-PC, EC, macrophages, and monocytes.

(**G**) The top ranked ligands in T-PC and N-PC as sender cells and EC as receiver cells according to *NicheNet*. Ligand activities were shown on the left. Expression of ligands were shown on the right. Bar color represents the ligand activity. Dot color represents the expression level. Dot size represents the fraction of population expressing the gene.

(**H**) The top10 ranked ligands and the corresponding receptors in intercellular communications between T-PC and EC as determined by *NicheNet*. The results were visualized with *bipartite* in R. Yellow represents ligand. Blue represents receptor. Pink represents the interaction between ligand and receptor.

Fig. S4.


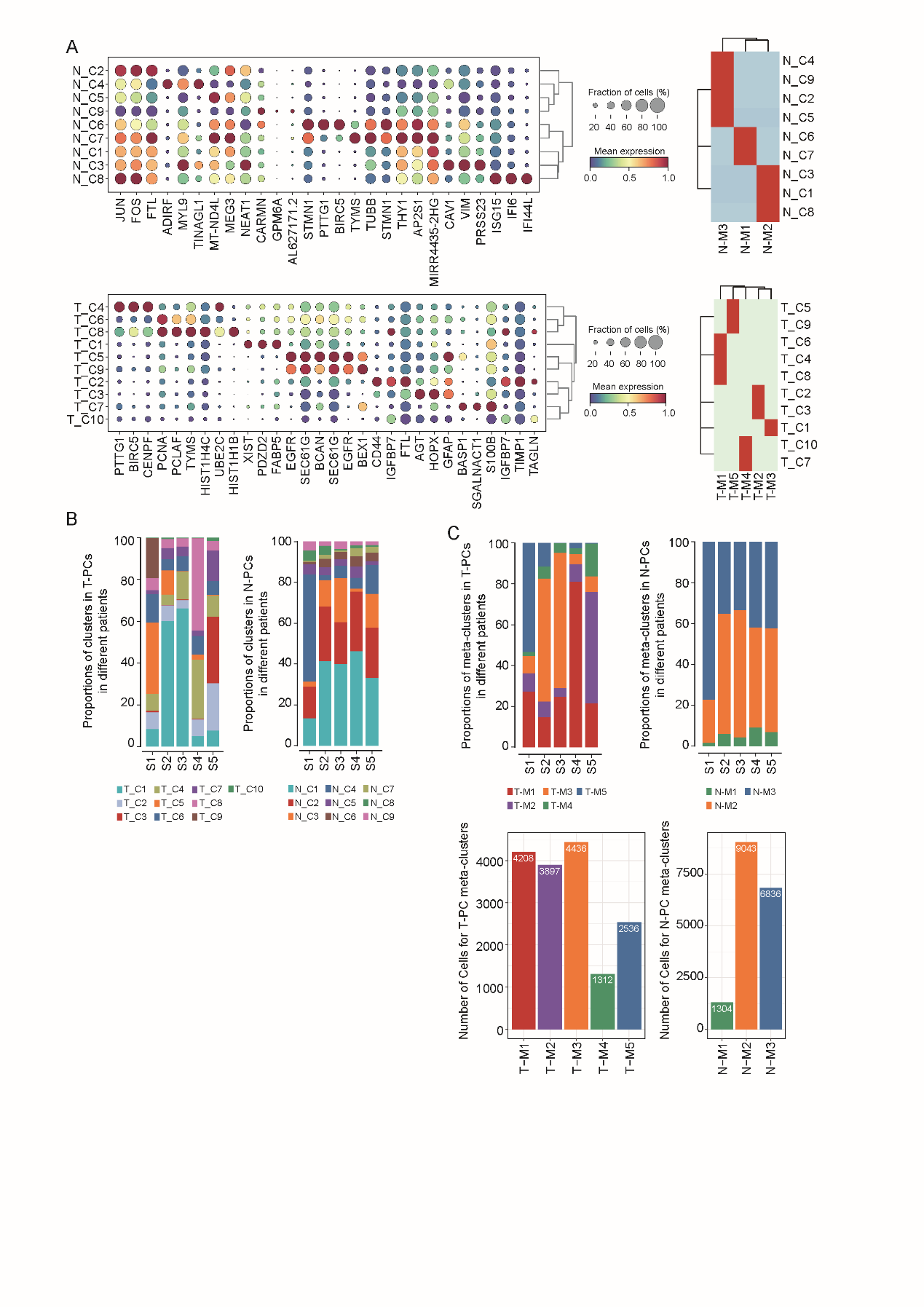


**Supplementary Fig S4.** **T-PC and N-PC are composed of distinctive heterogenous subpopulations**

1. Dot plots showing the top3 highly variable genes of the clusters identified in N-PC (upper left) and T-PC (lower left). On the basis of the highly variable genes, the clusters were further categorized into 3 meta-clusters for N-PC (upper right) and 5 meta-clusters for T-PC (lower right). Dot size represents the fraction of cluster expressing the gene. Dot color represents scaled gene expression.
2. Compositions of various clusters in N-PC (left) and T-PC (right) from different samples.
3. Compositions (left panels) and cell numbers (right panels) of various clusters in N-PC and T-PC from different samples.

Fig. S5.


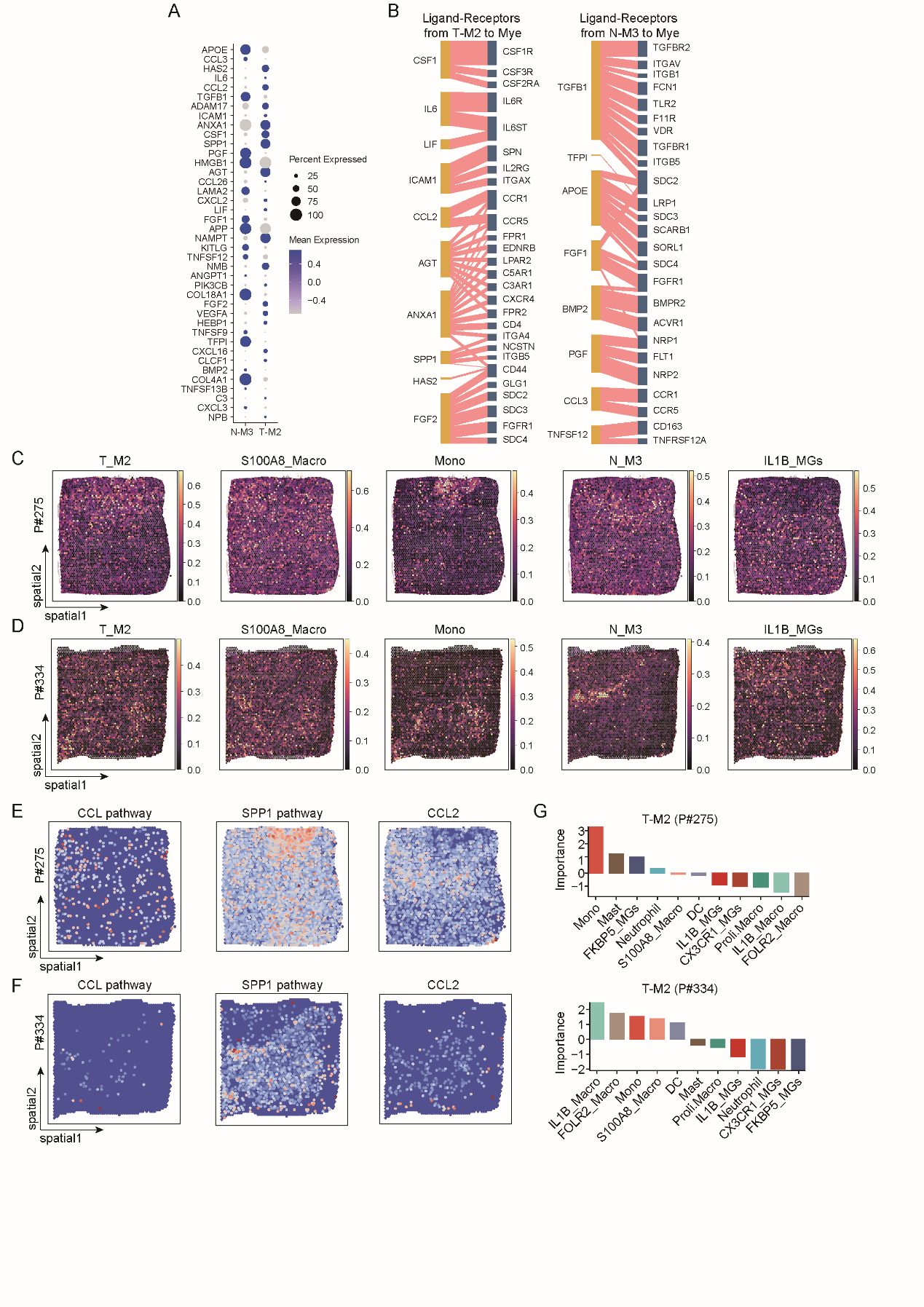


**Supplementary Fig S5. T-PC and N-PC contain different immuno-regulatory subpopulations**

(**A**) The top ranked ligands in T-M2 and N-M3 pericyte meta-clusters as sender cells and myeloid cells as receiver cells according to *NicheNet* analysis. Dot color represents the expression level. Dot size represents the fraction of population expressing the gene.

(**B**) The top10 ranked ligands and the corresponding receptors in intercellular communications between the indicated pericyte meta-clusters and myeloid cells as determined by *NicheNet* analysis. Yellow represents ligand. Blue represents receptor. Pink represents the interaction between ligand and receptor.

(**C** and **D**) Spatial mapping of the indicated cell populations in the public spatial transcriptomic data of the GBM samples P#275 (**C**) and P#334 (**D**) by using *Tangram*. Abundances of populations were evaluated through integration of scRNA-seq and spatial transcriptome. Color represents estimated cell abundance. Cell populations with similar spatial distributions were shown.

(**E** and **F**) Spatial mapping of the indicated signaling pathways and genes in the public spatial transcriptomic data of the GBM samples P#275 (**E**) and P#334 (**F**) by using *COMMOT*. The pathways and genes were from the intercellular signaling axes that may mediate the influence of T-M2 or N-M3 meta-clusters on myeloid cells. Color scale represents score of pathway or gene expression level. The pathways and genes showed spatial distributions similar to that of the T-M2 or N-M3 as in (**C**) and (**D**).

(**G**) Boxplots showing the levels of co-distribution of the T-M2 meta-cluster with the indicated immune populations in the public spatial transcriptomic data of GBM samples P#275 and P#334. Levels of co-distribution were evaluated with *mistyR* on the basis of the abundances and spatial positions of cell populations. Higher co-localization levels stand for more significant spatial correlation.

Fig. S6.


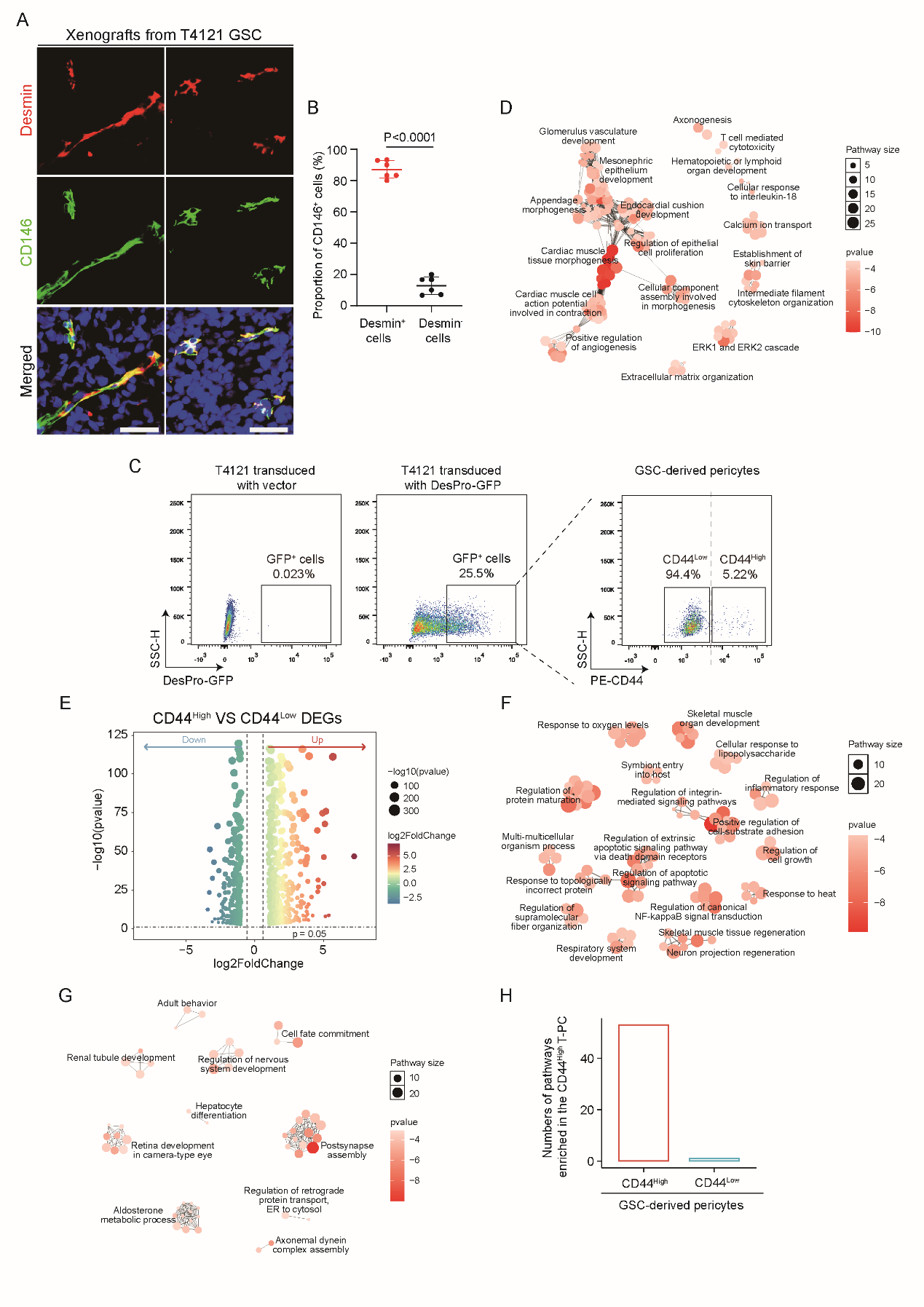


**Supplementary Fig S6. GSC-derived CD44^High^ pericytes resemble the immunoregulatory CD44^High^ T-PC**

(**A** and **B**) Representative images (**A**) and statistical quantification (**B**) of immunofluorescent analysis of Desmin (red) and CD146 (green) in mouse intracranial GBMs derived from T4121 GSCs. Frozen sections were immunostained with antibodies against Desmin and CD146, and counterstained with Hoechst to show nuclei (blue). Most CD146^+^ cells were positively stained for Desmin. Scale bar, 40 μm. (n = 5 sections for each group; unpaired two-tailed student’s t-test; mean ± s.d.)

(**C**) Representative flow cytometry plots of GFP^+^CD44^High^ cells in GBM cells differentiated from T4121 GSCs transduced with DesPro-GFP. Differentiation of T4121 GSCs transduced with DesPro-GFP or DesPro-vector were carried out through serum induction for 14 days. Cells were trypsinized and single cell suspensions were labeled with PE anti-human CD44 antibodies followed by flow cytometry sorting. Cells differentiated from GSCs transduced with DesPro-vector were used as negative control, while GFP^+^ and GFP^-^ cells were sorted from differentiated cells transduced with DesPro-GFP. Subsequently, from the GFP^+^ cells, CD44^High^ cells with high PE signals that were apart from the main cell cluster of CD44^Low^ cells with low PE signals in the cytometry scatter plot were sorted and collected as GSC-derived CD44^High^ and CD44^Low^ pericytes, respectively. Percentages of GFP^+^CD44^High^ cells in 10,000 cells were quantified.

(**D**) Visualization of pathway enrichment network of the CD44^High^ T-PC in the scRNA-seq data using *aPEAR*. DEGs in the CD44^High^ T-PC were inputted for GO enrichment by using *clusterprofiler*. The resultant pathways were clustered for the automatic generation of the enrichment networks. Dot color represents pvalue and dot size represents the pathway size.

(**E**) Volcano plot showing differentially expressed genes in GSC-derived CD44^High^ relative to CD44^Low^ pericytes in the UMI RNA-seq. Genes with p-value < 0.05 (Wilcoxon rank-sum test) and log2(fold change) > 0.5 were shown.

(**F** and **G**) Visualization of pathway enrichment networks of the GSC-derived CD44^High^ (**F**) and CD44^Low^ (**G**) pericytes in the UMI RNA-seq data using *aPEAR*. Differentially expressed genes in the GSC-derived CD44^High^ and CD44^Low^ pericytes were inputted for GO enrichment by using *clusterprofiler*. The resultant pathways were clustered for automatic generation of the enrichment networks. Dot color represents pvalue and dot size represents the pathway size.

(**H**) Boxplot showing the numbers of pathways enriched in the GSC-derived CD44^High^ and CD44^Low^ pericytes that were also enriched in the CD44^High^ T-PC in GBMs. The GSC-derived CD44^High^ pericytes and the CD44^High^ T-PC had more than 50 commonly enriched pathways, whereas the GSC-derived CD44^Low^ pericytes and the CD44^High^ T-PC had very few commonly enriched pathways.

Fig. S7.


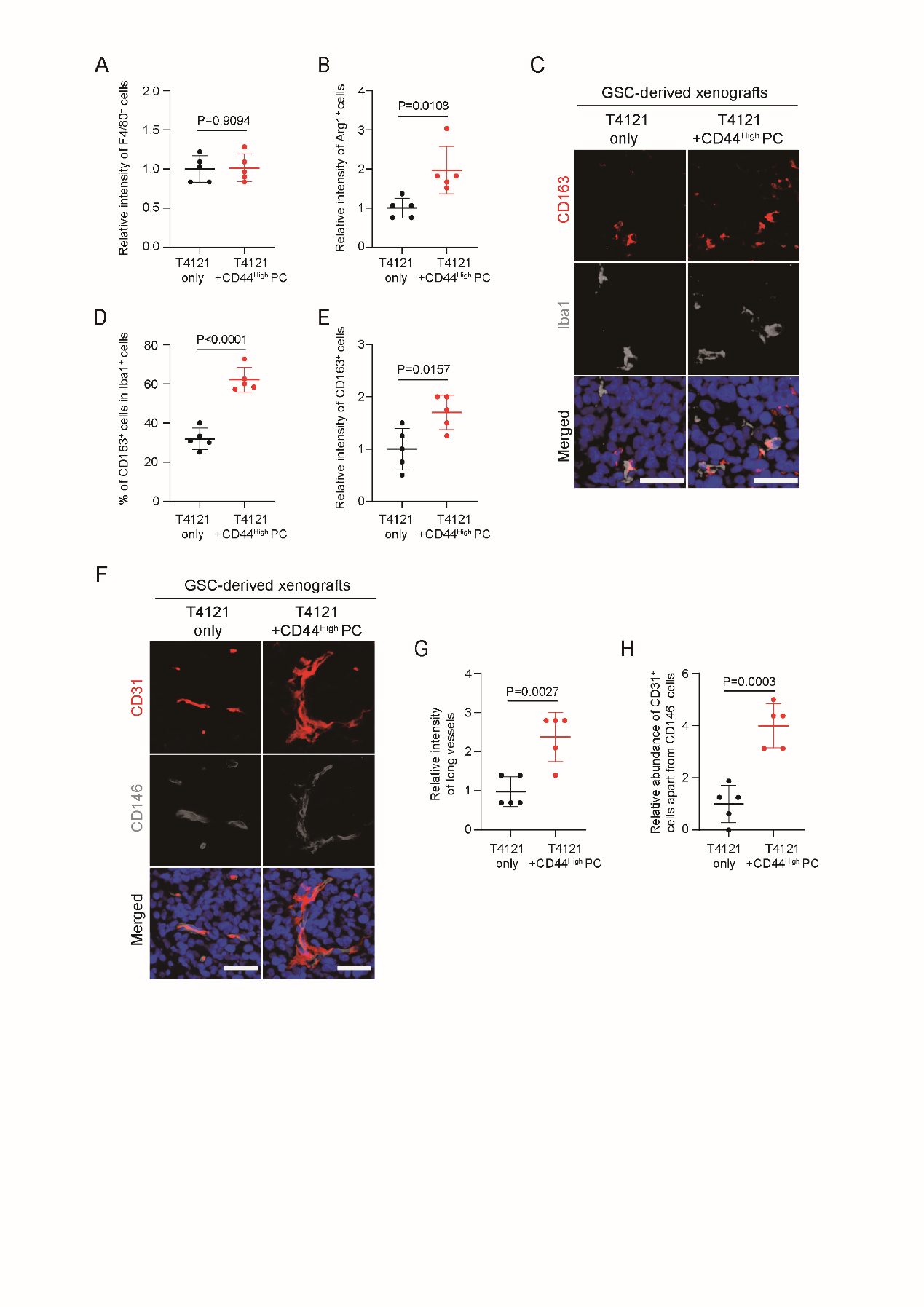


**Supplementary Fig S7. GSC-derived CD44^High^ pericytes promote GBM growth and M2-polarization of TAMs**

(**A** and **B**) Statistical quantification of immunofluorescent analysis of the pan-macrophage marker F4/80 (**A**) and the M2 macrophage marker Arg1 (**B**) in mouse intracranial GBMs derived from the T4121 GSCs with or without GSC-derived CD44^High^ pericyte co-implantation. The numbers of F4/80^+^ macrophages showed no apparent difference, whereas the numbers of the Arg1^+^ cells increased in xenografts derived from GSC and GSC-derived CD44^High^ pericyte co-implantation relative to those derived from GSC implantation. (n = 5 sections for each group; mean ± s.d.; two-tailed unpaired student’s t-test)

(**C** and **D**) Representative images (**C**) and statistical quantification (**D**) of immunofluorescent analysis of the M2 macrophage marker CD163 (red) and the pan-macrophage marker Iba1 (gray) in mouse intracranial GBMs derived from the T4121 GSCs with or without GSC-derived CD44^High^ pericyte co-implantation. Frozen sections were immunostained with antibodies against CD163 and Iba1, and counterstained with Hoechst to show nuclei (blue). Xenografts derived from GSC and GSC-derived CD44^High^ pericyte co-implantation had higher rates of M2 macrophages relative to those derived from GSC implantation. Scale bar, 40 μm. (n = 5 sections for each group; mean ± s.d.; two-tailed unpaired student’s t-test)

(**E**) Representative images of immunofluorescent analysis of CD31 (red) and CD146 (gray) in mouse intracranial GBMs derived from the T4121 GSCs with or without GSC-derived CD44^High^ pericyte co-implantation. Frozen sections were immunostained with antibodies against CD31 and CD146, and counterstained with Hoechst to show nuclei (blue). Scale bar, 40 μm.

(**F**) Statistical quantification of long vessels (> 100 μm) marked by CD31 on the basis of the immunofluorescent staining in (**E**). Xenografts derived from GSC and GSC-derived CD44^High^ pericyte co-implantation had more long vessels relative to those derived from GSC implantation. (n = 5 sections for each group; mean ± s.d.; two-tailed unpaired student’s t-test)

(**G**) Statistical quantification of CD31^+^ cells apart from CD146^+^ cells on the basis of the immunofluorescent staining in (**E**). Xenografts derived from GSC and GSC-derived CD44^High^ pericyte co-implantation had more CD31^+^ cells apart from CD146^+^ cells, which may represent the newly-formed immature vessels. (n = 5 sections for each group; mean ± s.d.; two-tailed unpaired student’s t-test)
